# Intratumoral IL12 mRNA administration activates innate and adaptive pathways in checkpoint inhibitor-resistant tumors resulting in complete responses

**DOI:** 10.1007/s00262-025-04105-0

**Published:** 2025-06-25

**Authors:** Jayalakshmi Lakshmipathi, Sreevidya Santha, Man Li, Yuping Qian, Simon F. Roy, Nadia Luheshi, Katerina Politi, Marcus Bosenberg, Jim Eyles, Viswanathan Muthusamy

**Affiliations:** 1 Yale Center for Precision Cancer Modeling, Yale Cancer Center, Yale School of Medicine, New Haven, CT USA; 2 AstraZeneca, Cambridge Biomedical Campus, Cambridge, CB2 0AA UK; 3 Departments of Pathology and Internal Medicine, Yale Cancer Center, Yale School of Medicine, New Haven, CT USA; 4 Department of Dermatology, Yale School of Medicine, New Haven, CT USA; 5 Department of Immunobiology, Yale School of Medicine, New Haven, CT USA; 6 Department of Medicine (Oncology), Yale School of Medicine, New Haven, CT USA; 7 Department of Pathology, McGill University, Montreal, Canada

**Keywords:** mRNA therapy, Intratumoral agent, Immunotherapy agent, Checkpoint resistance, Cytokine therapy

## Abstract

Despite the proven clinical activity of checkpoint inhibitors (ICIs) in several cancer indications, frequent occurrence of primary and secondary resistance reduces their overall effectiveness. Development of ICI resistance has been attributed mainly to genetic or epigenic alterations that affect the tumor antigen presentation machinery leading to diminished anti-tumor immune responses. There is an urgent need for new approaches which can either re-sensitize resistant tumors to the ICIs or engage alternate immune pathways to inhibit tumors. Intratumoral delivery of nanoparticle encapsulated murine IL12 (mIL12) mRNA induces powerful anti-tumor immune responses in murine tumor models, and the human version of this drug results in objective responses in patients with advanced disease. Here, we tested the efficacy of mIL12 mRNA as a single agent and in combination with anti-PD-L1 antibodies in ICI-sensitive Yummer 1.7 melanoma and MC38 colorectal murine tumors and in ICI resistant, β2-microglobulin (*B2M*) knockout versions of these models. mIL12 mRNA monotherapy was sufficient to cause complete responses (CRs) in ≥ 60% of both ICI-sensitive or -resistant Yummer 1.7 melanoma and MC38 colorectal carcinoma tumors. The mIL12 mRNA treatment resulted in potent upregulation of T_H_1 type cytokines and chemokines. A reduction in number of Tregs, increase in numbers and activation state of both cytotoxic T cells (CTLs) as well as tumor-associated macrophages (TAMs) was observed indicating enhanced anti-tumor, cell-based immune responses in the tumor microenvironment. This mIL12-induced concerted immune activation was associated with a robust killing and phagocytosis of tumor cells resulting in durable CRs. These observations suggest that intratumoral IL12mRNA therapy may benefit patients with ICI-resistant cancers.

**Supplementary Information:**

The online version contains supplementary material available at 10.1007/s00262-025-04105-0.

## Introduction

Immune checkpoint inhibitors (ICIs) have become the mainstay in cancer therapy in several indications, as they have the potential to promote durable and even curative responses [1–3]. Unfortunately, resistance to ICI develops in a significant proportion of patients, leading to disease progression and poor survival outcomes [4, 5]. Defects in antigen processing and presentation that blunt the anti-tumor immune responses are frequently encountered in ICI resistance [4, 6–9]. A common defect in antigen presentation is observed in the form of dysfunctional MHCI due to alterations which affect the B2-microglobulin (B2M) protein, a key component of the complex. Biallelic loss, loss of heterozygosity, point mutations and epigenetic silencing of the B2M gene that result in MHCI deficiency are frequently encountered across several human cancers and are thought to be contributory to ICI resistance [10–14].

Interleukin-12 (IL12) is a pro-inflammatory cytokine that influences both innate and adaptive immune cells by induction of IFNγ production [15]. Activation of IFNγ signaling is central to immune responses leading to tumor clearance and approaches that kick-start this pathway have shown promise in overcoming ICI resistance [16, 17]. Unfortunately, parenteral administration of IL12 results in unacceptable dose-limiting toxicities and limited efficacy [18–20]. A broad variety of adverse effects were observed including pyrexia, flu-like symptoms, nausea and diarrhea. Significant elevation of liver enzymes was observed serious hepatotoxicity. Broad hematologic adverse events included leukopenia, anemia, neutropenia and thrombocytopenia [21]. One approach to circumvent the poor tolerability of systemic IL12 therapies is to inject lipid nanoparticle encapsulated mRNA encoding recombinant human IL12 directly into the tumor microenvironment (TME) via an intratumoral (IT) injection. This would allow for controlled, transient production of IL12 in the vicinity of tumor allowing for hyperlocal, tumor-specific escalation of immune responses without triggering systemic effects. Preclinical experiments using this approach demonstrated that IT injection of lipid nanoparticle encapsulated mRNA encoding mouse IL12, potently inhibited the growth of murine tumors and showed additivity when combined with anti-PD-L1 in a syngeneic allograft model. In a first in human clinical trial, of an analogous drug, an IT delivered human IL12 encoding RNA altered the tumor microenvironment and induced objective responses in some patients [22].

Syngeneic cell lines engineered for ICI resistance have been developed and treated using systemic agents such as decoy-resistant IL18 or intratumorally administered TLR9 and IL2 agonists in a preclinical setting. While these agents were shown to elicit immune responses, the tumor inhibitory effects observed were unremarkable and only a small proportion of treated mice experienced CRs [23–25]. Since it has been already established that IL12 generates broad immune responses by activation of multiple effector pathways involving cytokines, chemokines, adaptive and innate immune cells [15, 26], we hypothesized that the intratumorally delivered mIL12 mRNA could be a safe and effective therapeutic agent which could engage available, unimpaired effector pathways to inhibit tumors resistant to ICIs.

In this work, we describe powerful anti-tumor responses resulting from mIL12 mRNA treatment of MHC class I-deficient, syngeneic allograft models, YUMMER 1.7 B2M knockout melanoma (Yummer B2M KO) and MC38 B2M knockout colorectal carcinoma (MC38 B2M KO), both of which were engineered by CRISPR knockout of the beta-2 microglobulin gene (B2M KO). These lines have been previously shown to form fast-growing tumors when engrafted in immune competent host mice and do not respond to systemic ICI therapy [25].

## Materials and methods

### Cell lines, drugs and reagents

Murine cell lines Yummer 1.7 wild type (WT), MC38 WT, Yummer B2M KO and MC38 B2M KO were grown in Opti-MEM medium (Gibco, 31,985-070) supplemented with 10% heat-inactivated fetal bovine serum (Sigma-Aldrich, F4135) and 1 × penicillin–streptomycin (15,140-122, Gibco), MEM non-essential amino acids (11,140-050, Gibco), L-glutamine (25,030-081, Gibco) and sodium pyruvate (11,360–070, Gibco).

The murine mIL12 mRNA and control non-coding mRNA (17,469 NTFIX) formulated in lipid nanoparticles (LNP) were from Moderna therapeutics. Anti-PD-L1 (mIgG1, Clone 80) and isotype mouse IgG antibody (NIP 228) were from AstraZeneca.

### Tumor implantation and in vivo treatments

All animal studies were conducted in accordance with protocol 2024–2028 approved by the Yale University Institutional Animal Care and Use Committee. Eight-week-old C57BL6/J mice (Jackson Laboratory, ME) were subcutaneously implanted in the right flank with 1 × 10^6^ MC38 WT or Yummer 1.7 WT cells for ICI-sensitive models-based experiments. 2 × 10^6^ MC38 B2M KO or Yummer B2M KO cells were implanted for ICI-resistant tumor model studies. When the tumors were palpable (~ 50–100 mm^3^), the mice were allocated to the following four treatment groups: (1) control treatment with non-coding mRNA and isotype antibody, (2) mIL12 mRNA monotherapy, (3) anti-PD-L1 monotherapy and (4) combination therapy with mIL12 mRNA and anti-PD-L1. Treatment with drugs or their control counterparts were administered at the same time for each model tested. The mIL12 and non-coding control mRNAs were dosed once, 0.5 µg in a 50 µL volume directly administered into the tumor core via an intratumoral injection. The anti-PD-L1 or isotype control antibodies (250 µg/animal) were administered intraperitoneally every 3 days for four cycles. Tumor dimensions were measured daily after treatment was started using digital calipers. Tumor volumes were calculated using the formula 0.5 × length × width^2^. The animals were euthanized when the endpoint tumor volume of 1 cm^3^ was reached. Mice with CRs were observed for 70 days after implantation and then subjected to one round of rechallenge with 2 × 10^6^ cells (ICI sensitive, WT models) or 4 × 10^6^ cells (B2M KO models) injected into the opposite (left) flank and observed for further 30 days.

The wild-type model studies were performed with five animals per group as ICI effects in these models have been previously studied. For the ICI resistance models, which are central to the study, the drug efficacy studies were carried out using 10 mice per group for improved precision. Pharmacodynamic studies were performed separately using five mice per group; with serum collected for cytokine analyses, and tumor tissues for immunohistochemistry and flow cytometry analysis.

### Cytokine analysis of serum from treated mice

Mice allocated to all treatment groups were euthanized 8 days after the beginning treatment. Blood was collected during the necropsy, allowed to clot for 30 min, and serum was prepared. The serum was stored frozen at − 80 °C until the cytokine assays were conducted. The levels of 44 cytokines were quantified using the Mouse Cytokine/Chemokine 44-Plex Discovery Assay (Eve Technologies, MD44). The analyzed cytokines include CCL11, EPO, CCL21, CX3CL1,CSF3, CSF2, IFNB1, IFNG, IL1A, IL1B, IL2, IL3, IL4, IL5, IL6, IL7, IL9, IL10, IL11, IL12B, IL12p70, IL13, IL15, IL16, IL17A, IL20, CXCL10, CXCL1, LIF, LIX, CCL2, CCL12, CSF1, CCL22, CXCL9, CCL3, CCL4, CXCL2, CCL20, CCL19, CCL5, CCL17, TNF, VEGFA and TIMP1.

### Flow cytometric analysis of tumor-infiltrating cells

Single-cell suspensions were prepared from tumor fragments using a combination of enzymatic and mechanical dissociation. The harvested tissues were minced in media and incubated at 37 °C with a cocktail of 0.1 mg/mL collagenase IV (C5138, Sigma-Aldrich), 0.1 mg/mL DNase I (10,104,159,001, Rohe) and 5 mM of CaCl_2_ (793,639, Sigma-Aldrich) for 20 min. The samples were passed through a 40-μm strainer to remove large debris. Tumor cells were resuspended in FACS buffer (PBS with 2% BSA, 5% FBS) at a concentration of 5 × 10^6^ cells/mL for staining. Non-specific antibody binding was blocked using anti-mouse CD16/CD32 Fc block solution (Cat. No. 101320, Biolegend) as per manufacturer’s protocol. Cells were incubated with fluorophore-conjugated antibodies (Supplementary Table 1) in FACS buffer in multiplexed reactions for 20 min at room temperature. After antibody staining, cells were washed in FACS buffer, and flow cytometry was performed using Attune NxT Flow Cytometer (Thermo Fisher Scientific). Data analysis was performed using FlowJo software (v10.10).

### Histological analysis of the tumor microenvironment

The harvested tumors were fixed in 10% formalin, embedded in paraffin and sectioned into 5 µm slices. Subsequently, the sections were stained for histology with Hematoxylin and Eosin (H&E) and with specific antibodies targeting CD45, CD3 and F4/80 for immunohistochemistry (IHC) at Yale Pathology Tissue Services core, according to the standard protocols. For mitotic and pyknotic event quantification, wherever tumor material was available, five high magnification (original magnification × 400) optical fields per H&E section were evaluated by a board-certified pathologist (SFR), and the counts for each field were scored blinded to the therapy or control status.

### Statistical analysis

Data analyses were performed using the ‘Built-in Statistical Analysis’ suite of GraphPad Prism software (v10.3.1) (GraphPad Software, La Jolla, CA, USA). Specifically, Log-Rank (Mantel–Cox) tests were performed to analyze the Kaplan–Meier survival data from the drug efficacy studies. Multiple unpaired t-tests (Welch t-tests) were performed on flow cytometry data to verify significant changes in infiltrating cells in drug-treated tumors compared to control treated tumors.

## Results

### mIL12 mRNA treatment results in CRs and enhances PD-L1 efficacy in ICI-responsive tumor models

We first investigated the efficacy of mIL12 mRNA in a monotherapy and combination setting along with PD-L1 antibody treatment in the well characterized, Yummer 1.7 melanoma and the MC38 colorectal tumor cell lines allografted subcutaneously into flanks of wild-type C57BL/6 mice.

**Fig. 1:**
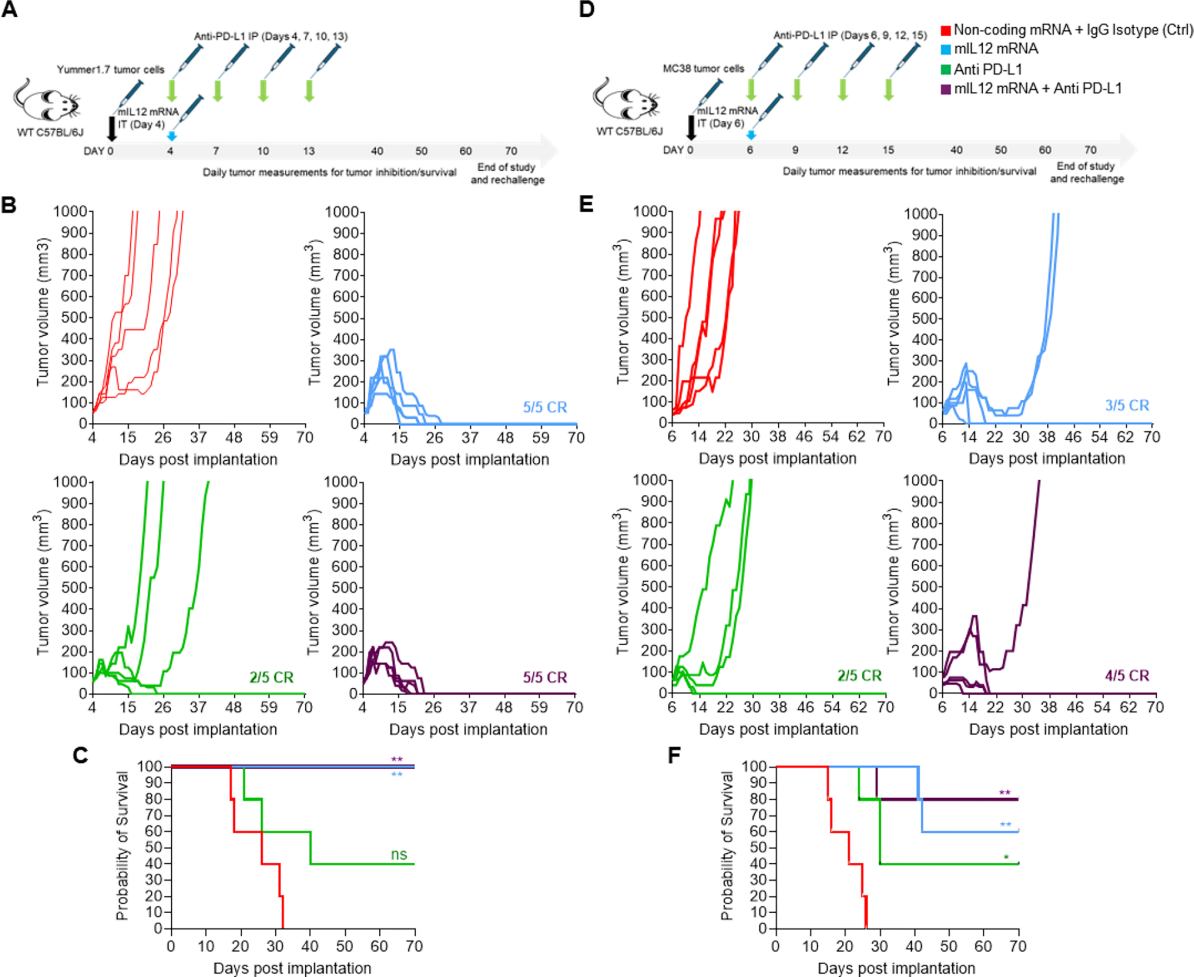
mIL12 mRNA monotherapy and combination with anti-PD-L1 results in complete response in both Yummer 1.7 and MC38 syngeneic allografted tumors. **A** Schematic showing the treatment strategy and timeline adopted in the Yummer 1.7 tumor model. **B** Spaghetti plots of tumor volume measurements of individual Yummer 1.7 tumor-bearing mice over time upon treatment showing inhibition and response rates. **C** Survival of Yummer 1.7 tumor-bearing mice in the different treatment groups, **D** schematic showing the treatment strategy and timeline adopted in the MC38 tumor model, and **E** spaghetti plots of tumor volume measurements of individual MC38 tumor-bearing mice over time upon treatment showing tumor inhibition and response rates. **F** Survival of MC38 tumor-bearing mice in the different treatment groups. *P* values are from Log-Rank (Mantel–Cox) test comparing treatment arms to control treatment *P ≤ 0.01, **P ≤ 0.001 and ***P ≤ 0.0001, ns—not significant

Administration of a single IT injection of mIL12 mRNA into palpable, tumor masses resulted in CRs in all Yummer 1.7 tumor-bearing mice (Fig. 1A and B). Signs of tumor inhibitory effect of mIL12 mRNA treatment were observable in a week after administration. The first CRs were attained in 2 weeks, and all mice were cured within 4 weeks of treatment initiation (Fig. 1B). Anti-PD-L1 treatment resulted in CRs in 2/5 mice but due to lack of responses in other mice in this group, did not translate to statistically significant survival advantages compared to the control arm (Fig. 1C). Combination treatment with both mIL12 mRNA and anti-PD-L1 resulted in CRs in all treated mice (Fig. 1B). The transient growth of existing tumors after treatment and time taken to observe complete responses in all individuals were decreased in the combination group compared to the mIL12 mRNA monotherapy group suggesting improved efficacy of the combination treatment (Fig. 1B). CRs in all mice remained durable for over 4 weeks after disappearance of the tumors (Fig. 1C). Rechallenge of the cured mice with implantation of 2 × tumor cells on the opposite flank did not result in tumor establishment.

We observed tumor growth inhibition (TGI) in all the MC38 tumor-bearing mice treated with mIL12 mRNA (Fig. 1D and E). CRs occurred in 60% (3/5) and partial responses (PRs) were observed in the remaining mice within 2 weeks upon treatment with mIL12 mRNA alone. The PRs were associated with significant survival advantages over control treatment (Fig. 1F). Therapy with anti-PD-L1 resulted in CRs in 40% (2/5) of mice, and PRs in another 40% of the mice with improved survival compared to control treatment (Fig. 1E and F). In the combination treatment setting, all animals exhibited responses, and the complete response rate increased to 80% (4/5) showing a trend of improved effect compared to monotherapy groups. The tumor in one remaining individual mouse showed significantly slower growth and survival benefit (Fig. 1E and F). CRs remained durable for over 6 weeks after occurrence of cures (Fig. 1E), and rechallenge of the cured mice with 2 × tumor cells on the opposite flank did not result in tumor establishment.

### mIL12 mRNA monotherapy leads to durable CRs in ICI-resistant tumor models

We validated the MHCI expression status on the surface of Yummer and MC38 B2M KO cells by flow cytometry analysis. After stimulation with IFNγ, surface MHCI expression was lower than corresponding WT cell lines (Supplementary Fig. S1). Allografted tumors were established using the B2M KO lines in wild-type C57BL/6 mice, and these tumor models were utilized to evaluate the efficacy of mIL12 mRNA in experiments analogous to the unmodified Yummer 1.7 and MC38 parental tumor models.

**Fig. 2:**
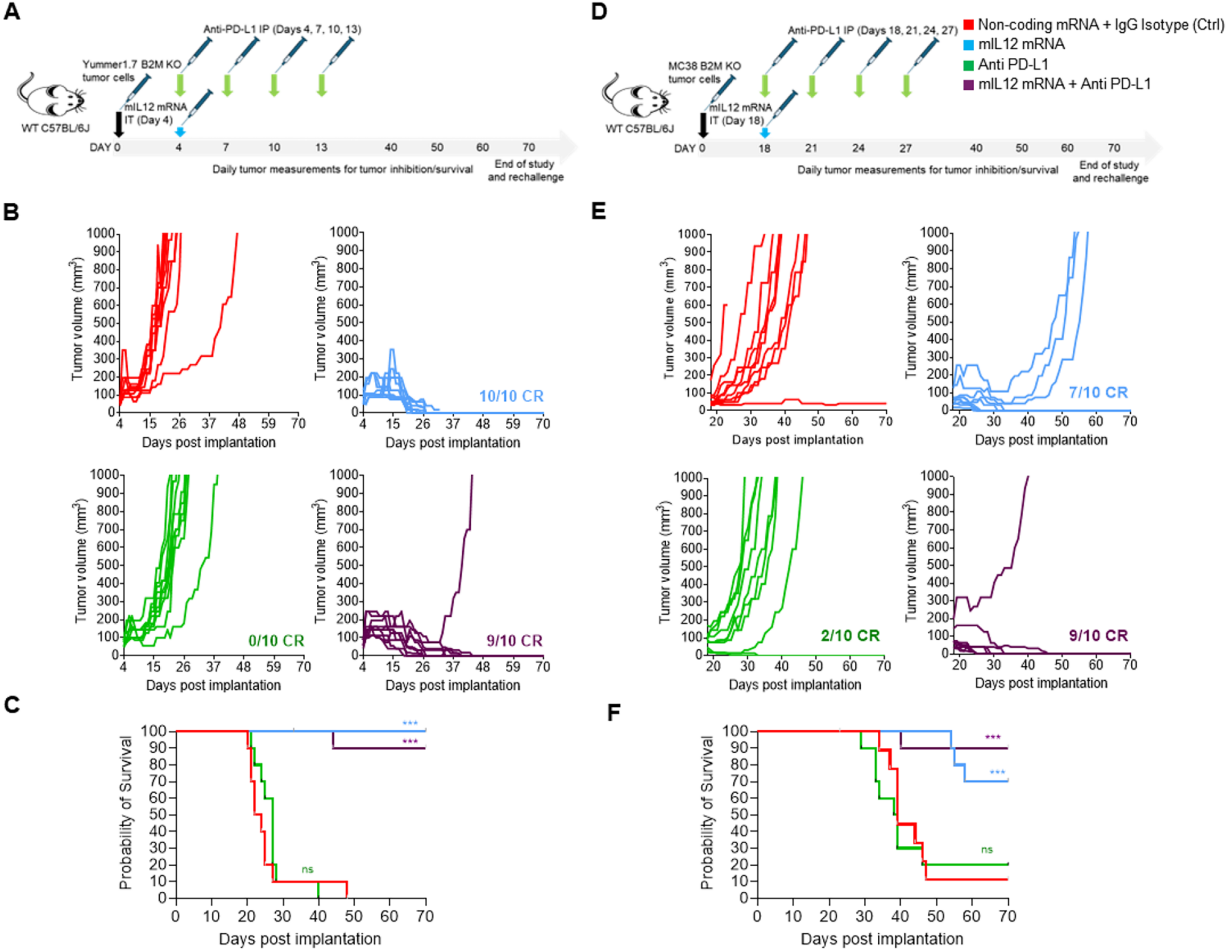
mIL12 mRNA monotherapy and combination with anti-PD-L1 results in complete response in Yummer B2M KO and MC38 B2M KO syngeneic allografted tumors. **A** Schematic showing the treatment strategy and timeline adopted in the Yummer B2M KO tumor model, **B** spaghetti plots of tumor volume measurements of individual Yummer B2M KO tumor-bearing mice over time upon treatment showing inhibition and response rates. **C** Survival of Yummer B2M KO tumor-bearing mice in the different treatment groups, **D** schematic showing the treatment strategy and timeline adopted in the MC38 B2M KO tumor model, and **E** spaghetti plots of tumor volume measurements of individual MC38 B2M KO tumor-bearing mice over time upon treatment showing tumor inhibition and response rates. **F** survival of MC38 B2M KO tumor-bearing mice in the different treatment groups. *P* values are from Log-Rank (Mantel–Cox) test comparing treatment arms to control treatment *P ≤ 0.01, **P ≤ 0.001 and ***P ≤ 0.0001, ns—not significant

In the Yummer B2M KO tumor model, IL12 mRNA monotherapy resulted in CRs in all (10/10) treated animals. Along expected lines, no responses were observed in the anti-PD-L1 treatment group in this model. CRs were observed in 9/10 animals in the combination therapy group. Slower tumor growth rates and earlier CRs were observed, suggesting a mild enhancement of TGI effect in this group. In one individual in this group, the implanted tumor grew rapidly after initially shrinking for 4 weeks suggesting occurrence of stochastic events in this otherwise responding tumor (Fig. 2A and B). Inhibitory effects of mIL12 mRNA treatment on tumor size and growth were observable in 1 week after the IT injection, CRs started to occur in 2 weeks and within 4 weeks after treatment, the responding individuals were cured with no signs of remaining tumor (Fig. 2A and B).

The survival rate in animals that underwent mIL12 mRNA therapy was 95% after 2 months including the combination arm, despite the outlier mouse (Fig. 2C). The CRs achieved by mIL12 mRNA treatment were durable, and the cured animals remained tumor free after rechallenging with 2 × numbers of Yummer B2M KO cells implanted into the opposite flank.

Monotherapy with mIL12 mRNA in the MC38 B2M KO tumor model led to TGI effects in all animals in the group culminating in CRs in (7/10) and PRs in 3/10 animals (Fig. 2D and E). The anti-tumor effect of mIL12 mRNA treatment occurred early, and tumor volume differences between mIL12 mRNA and vehicle arms were discernible 1 week after IT injection (Fig. 2E). Single-agent anti-PD-L1 treatment induced in CRs in 2/10 animals but this did not result in a statistically significant survival benefit compared to the control arm (Fig. 2F). Treatment with both mIL12 mRNA and PD-L1 resulted in 9/10 CRs indicating a modest additive TGI effect may have been afforded by the two-drug combination. Survival was significantly improved (≥ 70% at 2 months) in the groups treated with mIL12 mRNA. Median survival could not be determined in groups treated with mIL12 mRNA due to CRs, and the moderately improved survival in the combination arm was not statistically significant compared to mIL12 mRNA alone treatment (Fig. 2F).

### mIL12 mRNA treatment upregulates T_H_1 cytokines in B2M KO tumor models

To understand the immune mechanisms activated by mIL12 mRNA that underpin the strong tumor inhibition despite deficient antigen presentation in both Yummer 1.7 and MC38 B2M KO tumors, we performed pharmacodynamic studies by replicating the study design and treatment regime as for efficacy experiments above, but the studies were terminated early when the tumors were in an active response state. The study was ended and tissues for analyses harvested from all mice when two consecutive, daily measurements indicated tumor shrinkage or stabilized tumor volume in > 2/5 mice in the treatment groups. In both Yummer 1.7 and MC38 B2M KO models, the first signs of active tumor inhibition were observed 8 days after treatment with mIL12 mRNA (Supplementary Fig. S2).

**Fig. 3:**
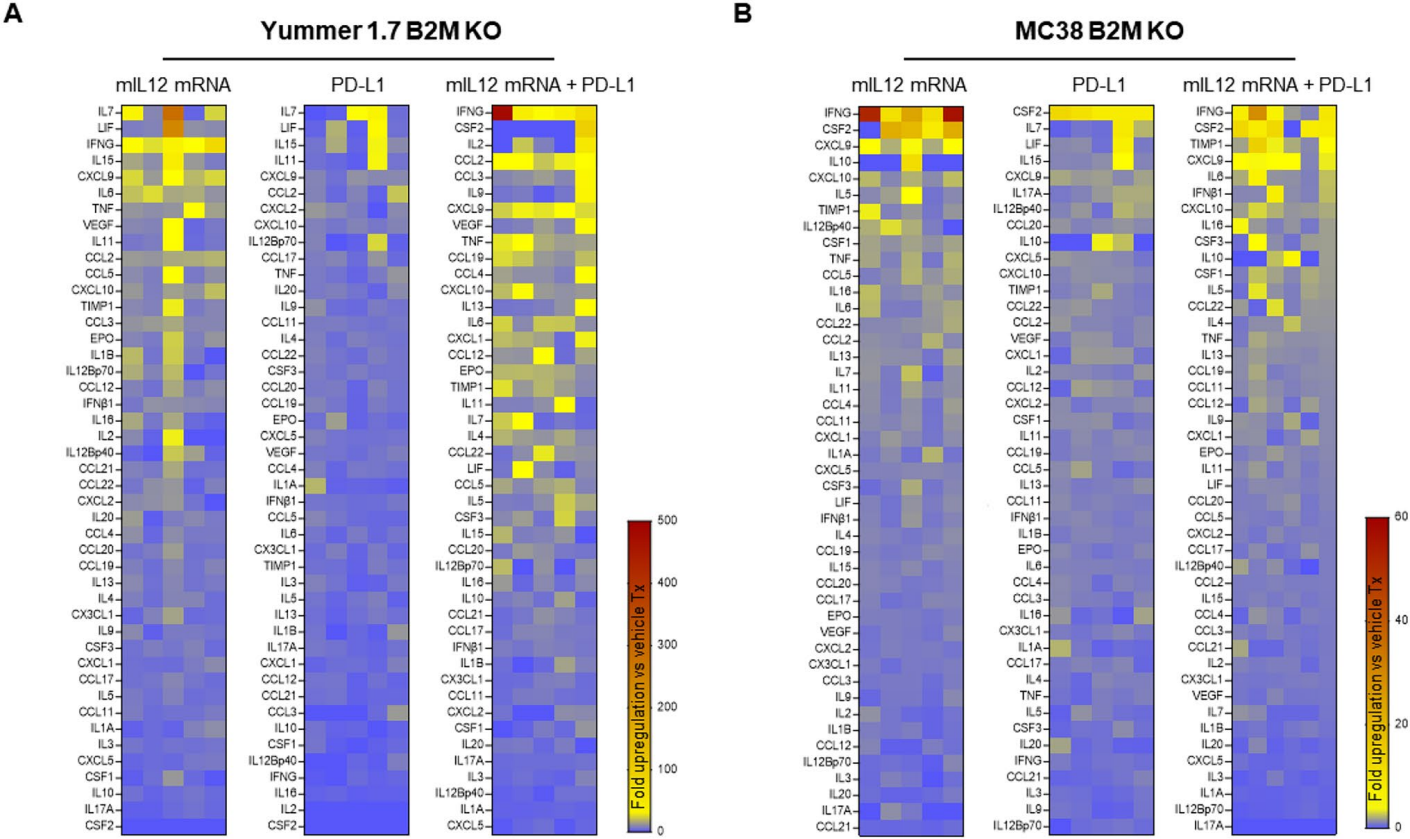
Multiplexed cytokine/chemokine analyses showing upregulation of T_H_1 type responses induced by mIL12 mRNA treatment. **A** Heat-map showing fold-changes in serum cytokine level changes in Yummer B2M KO tumor-bearing mice following drug treatment relative to control treatment. **B** Heat-map showing fold-changes in serum cytokine level changes in MC38 B2M KO tumor-bearing mice following drug treatment relative to control treatment. The heat-maps of each treatment group are rank listed in descending order with the greatest average increases 1 week after drug administration depicted at the top of the list. Each column represents serum from an individual mice in the treatment group. Each square represents fold difference of the indicated cytokine in one treated individual mouse relative to average levels in five control treated mice

Multiplexed analysis of circulating factors in the serum derived from the mIL12 mRNA both Yummer 1.7 and MC38 B2M KO tumor-bearing mice revealed elevated levels of cytokines responsible for major aspects necessary for successful anti-tumor responses. Comparison of cytokine levels in the IL12 treatment groups to levels in the control treatment group suggests pharmacologic modulation of similar pathways in both models (Fig. 3). The top upregulated cytokines, in a mean expression ranked list, that were changed relative to average levels in untreated tumors included the classic T_H_1 response cytokines IFNγ, TNFα; pro-inflammatory cytokines IL6, IL16; chemokines that promote migration of immune cells, CXCL9 (MIG), CXCL10 (IP-10), CCL5 (RANTES), CCL2 (MCP-1) and the immune cell proliferation promoting cytokine, IL7 (Fig. 3).

In stark contrast, monotherapy with anti-PD-L1 failed to increase T_H_1/pro-inflammatory cytokines in either model (Fig. 3). Combining the mIL12 mRNA and anti-PD-L1 treatments amplified the magnitude of upregulation and broadened types of cytokine/chemokines induced by the individual monotherapies (Fig. 3). Finally, it was reassuring to note that the intratumoral mIL12 mRNA administration did not result in high levels of circulating IL12 in the serum of the treated mice (Fig. 3). This is consistent with intended localized activity in the immediate tumor microenvironment and explains the absence of adverse reactions in these mouse models that occur in the case of systemic administration of IL12.

### mIL12 mRNA treatment activates innate immune cells in the TME of B2M KO tumors

**Fig. 4:**
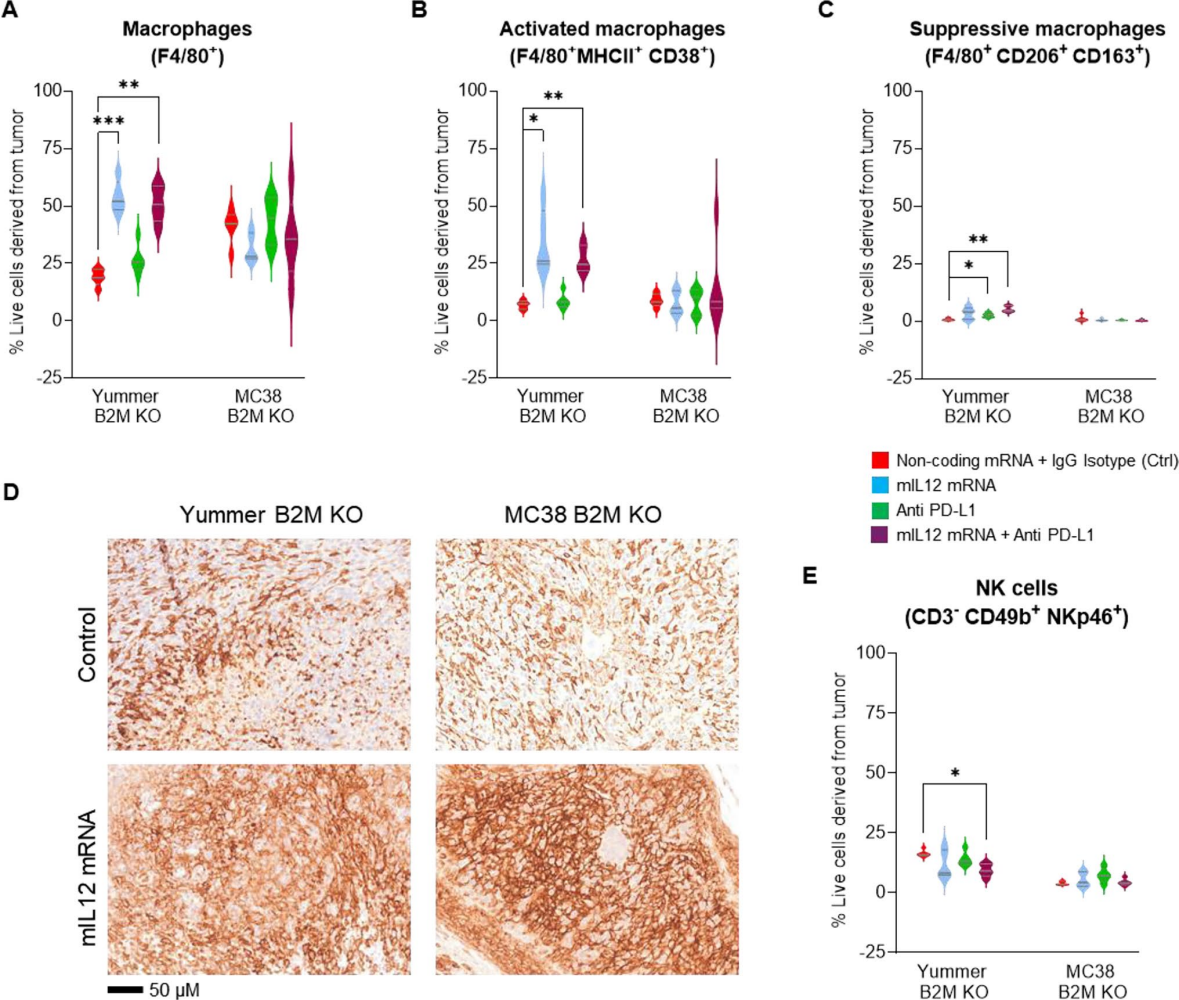
Innate immune response induced by mIL12 mRNA monotherapy in the MC38 B2M KO and Yummer B2M KO syngeneic allograft models. **A** Flow cytometry analyses data showing a significant increase in the numbers of F4/80^+^ macrophages in Yummer B2M KO tumors treated with mIL12 mRNA. **B** Analysis of flow cytometry data showing increased numbers of activated F4/80^+^MHCII^+^CD38^+^ immune activating type macrophages in Yummer B2M KO tumors treated with mIL12 mRNA. **C** Flow cytometry data analyses showing increase of tumor suppressive F4/80^+^MHCII^+^CD38^+^ macrophages in Yummer B2M KO tumors treated with mIL12 mRNA. D, IHC staining with F4/80 antibodies showing regionally increased TAM infiltration in both mIL12 mRNA-treated Yummer and MC38 B2M KO tumors. E, Flow cytometry analyses data showing infiltration status of CD3-CD49b^+^NKp46^+^NK cells in Yummer B2M KO and MC38 B2M KO tumors. Lines on the Box and violin plots indicate median and quartiles. Only significant differences (from t-test) are displayed—*P ≤ 0.01, **P ≤ 0.001 and ***P ≤ 0.0001

Flow cytometry analysis revealed that the control treated Yummer B2M KO tumors were infiltrated by approximately 13–19% F4/80^+^ TAMs. Compared to this baseline, a greater than threefold increase in numbers occurred resulting in 47–65% tumor cells being comprised of TAMs after mIL12 mRNA treatment (Fig. 4A). A vast majority of the F4/80^+^ TAMs in the mIL12 mRNA-treated tumors were found to be co-expressing MHCII and CD38 markers suggesting an immune activating phenotype. The average numbers of these type of macrophages were increased over fourfold compared to control tumors (Fig. 4B, Supplementary Table 2). In contrast, the numbers of the MHCII^+^CD38^+^ TAMs were not significantly changed in tumors that underwent PD-L1 monotherapy. Immune suppressive TAMs, characterized by expression of CD206 and CD163 markers, were a minority and occurred in approximately ninefold less numbers compared to MHCII^+^CD38^+^ TAMs (Supplementary Table 2). The numbers of immune suppressive TAMs appeared to mildly increase in treated Yummer B2M KO tumors (Fig. 4C, Supplementary Table 2). However, ratio between the immune activating and suppressive type macrophages was increased in some individuals treated with mIL12 mRNA suggesting TAM polarization in these tumors (Supplementary Fig. S3, Supplementary Table 2). The macrophage distribution in tumors treated with the combination was similar to mIL12 mRNA monotherapy tumors (Supplementary Table 2) suggesting that the IL12 treatment was primarily responsible for inducing the TME to an active state.

In the MC38 B2M KO model, the total numbers of TAMs infiltrating the control treated tumors ranged between 29 and 49% tumors and were not significantly changed in the mIL12 mRNA-treated tumors (26–41%). Immune suppressive type macrophage numbers were present in small numbers and their numbers decreased in many tumors that received mIL12 mRNA treatment (Supplementary Table 3). A similar effect was also noticed in tumors from the anti-PD-L1 monotherapy treatment group. The ratio of immune activating TAMs to suppressive TAMs showed a trend toward increase in all the treatment groups (Supplementary Fig. S3, Supplementary Table 3). In this model, anti-PD-L1 treatment appears to be contributory to the IL12-mediated enhancement of an immune-activated TME and could explain the modest improvement in efficacy observed with combination therapy. IHC analysis with F4/80 antibody staining of the control treated B2M KO tumor sections from both models showed a dense and even distribution of TAMs throughout the tumor. The mIL12 mRNA-treated tumor sections from both models showed intensely stained areas within the tumor with more cells stained per field than observed in the control tumor sections representing the focal increases in numbers of TAMs in the TME (Fig. 4D).

Flow cytometry analysis of the tumors for NK cells by staining with CD49b and NKp46 markers showed that NK cells were increased in 1/5 treated individuals in the Yummer B2M KO tumors treated with mIL12 mRNA or PD-L1 monotherapy. Overall, the changes were non-significant across all treatment groups. On the other hand, combination therapy appeared to mildly decrease NK cell numbers (Fig. 4E, Supplementary Table 2). On the other hand, in the MC38 B2M KO tumors, there were twofold or greater increases in NK cell numbers in 2/5 mice in both monotherapy groups. A trend toward modest increases in NK cell population was observed across the treatment groups (Fig. 4E, Supplementary Table 3).

### mIL12 mRNA therapy enhances CTL infiltration and decreases certain CD4^+^ subtypes in the TME of B2M KO tumors

**Fig. 5:**
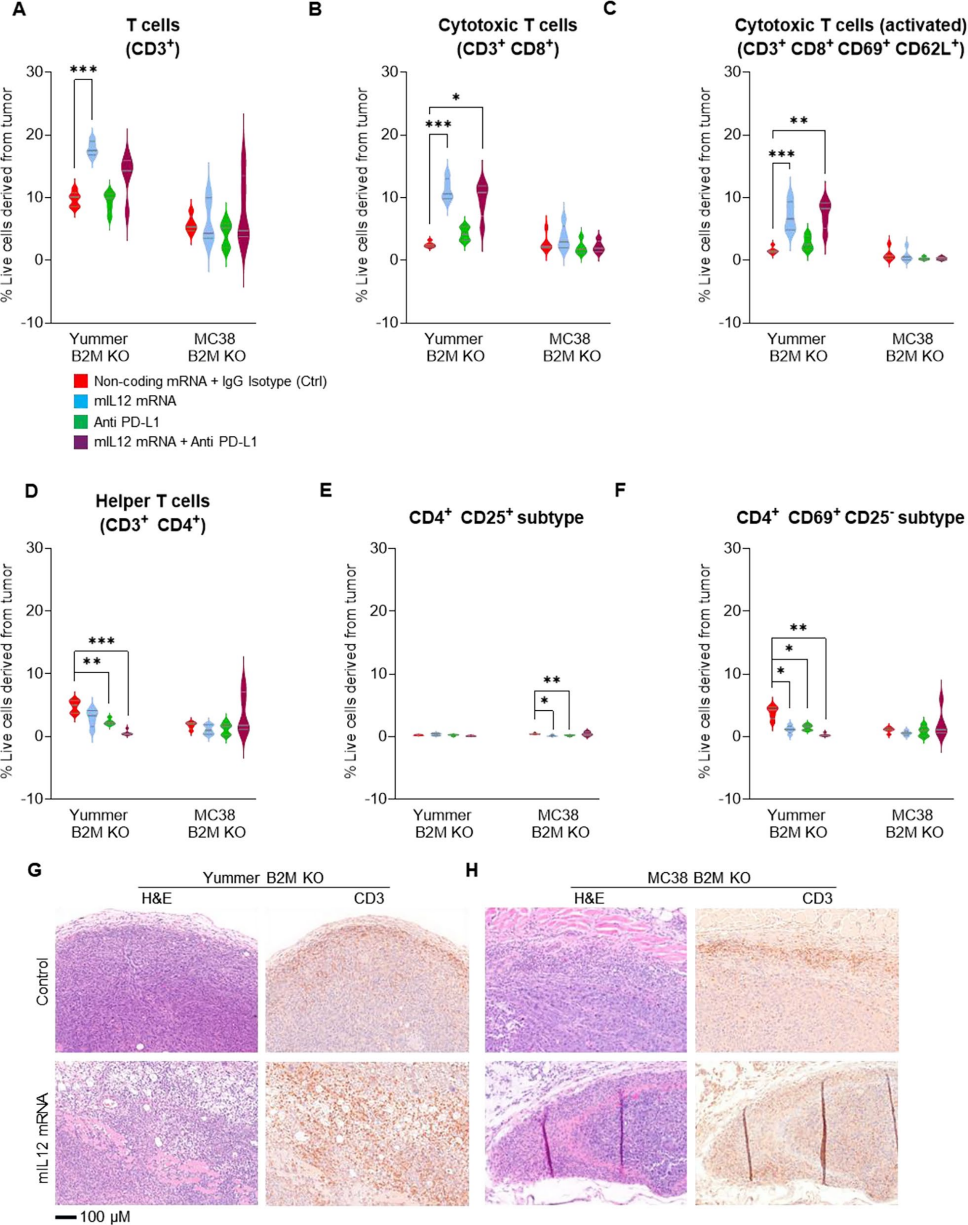
Increased infiltration of T cells induced by mIL12 mRNA monotherapy in the MC38 B2M KO and Yummer B2M KO syngeneic allograft models. **A** Flow cytometry analyses data showing significant increase in CD3^+^ T cells in Yummer B2M KO tumors treated with mIL12 mRNA and **B** flow cytometry analyses showing several fold increases in CD8^+^ cytotoxic T cells in Yummer B2M KO tumor treated with mIL12 mRNA. **C** Flow analyses showing increased numbers of activated CD8^+^CD69^+^CD62L^+^cytotoxic T lymphocytes in Yummer B2M KO tumors treated with mIL12 mRNA. **D** Flow analyses showing decreased CD4^+^ T cells in Yummer B2M KO and unchanged in MC38 B2M KO tumors treated with mIL12 mRNA. **E** Flow analyses showing decreased classic CD4 + CD25 + Tregs in MC38 B2M KO tumors. **F** Flow analyses showing decreased CD4^+^CD69^+^CD25^−^Treg cells in Yummer B2M KO tumors. **G** Yummer B2M KO histology, H&E showing uninhibited cell growth in control treated tumor, tumor cell death, inflammatory cells and pyknotic events in mIL12 mRNA-treated tumors; IHC with CD3 staining showing moderate inflammatory infiltrate limited to the peritumoral area in control tumors and robust T-cell infiltration, tumor cell clearance in mIL12 mRNA-treated tumors. **H**, MC38 B2M KO histology, H&E showing tumor cell growth in control treated and cell death, pyknotic events in mIL12 mRNA-treated tumors; IHC with CD3 staining showing limited, peritumoral *T* cell in control tumors and robust *T* cell infiltration, tumor clearance in mIL12 mRNA-treated tumors. Lines on the Box and violin plots indicate median and quartiles. Only significant differences (from t-tests) are displayed—*P ≤ 0.01 **P ≤ 0.001 and ***P ≤ 0.0001

Flow cytometry analysis revealed significantly increased numbers of CD3^+^ T cells in mIL12 mRNA-treated Yummer B2M KO tumors compared to control treated tumors (Fig. 5A). A majority of the CD3^+^ cells were CD8^+^ CTLs (Supplementary Table 2) in the mIL12 mRNA-treated tumors, and their average numbers were increased by over fourfold compared to the average numbers in control tumors (Fig. 5B). The CTLs expressing activation markers CD69 and CD62L were also increased by greater than fourfold in the mIL12 mRNA-treated tumors compared to control tumors indicating an active CTL-mediated anti-tumor response environment (Fig. 5C). The numbers of infiltrating CD4^+^ cells were significantly decreased across the treatment groups compared to numbers in the control tumors (Fig. 5D, Supplementary Table 2). A vast majority of these CD4^+^ cells (66–94%) could be classified either as CD4^+^CD25^+^ or CD4^+^CD69^+^CD25^−^ based on surface marker expression (Supplementary Table 2). In this model, CD4^+^CD69^+^CD25^−^ cells were the dominant population with about 80% of the CD4 cells falling in this category while the CD4^+^CD25^+^s constituted about 6% of the T helper cells (Supplementary Table 2). Treatment with mIL12 mRNA or anti-PD-L1 singly or in combination significantly reduced the population of the CD4^+^CD69^+^CD25^−^ type cells suggesting that this subpopulation of T helpers could be regulatory cells with a suppressive function in this model (Fig. 5 E and F, Supplementary Table 2).

In the MC38 B2M KO tumors receiving mIL12 mRNA monotherapy, CD3^+^ T-cell numbers were modestly increased in only 2/5 tumors relative to the average numbers in the control treated tumors. The numbers of CD8^+^ CTLs were increased in 4/5 of these tumors (Supplementary Table 3). However, the CD8^+^ cells were not highly positive for CD69 and CD62L; reflecting a less activated phenotype than detected in similarly treated Yummer B2M KO tumors. The CD4^+^ cell numbers were not changed significantly but trended toward a decrease in monotherapy and increase the combination treatment (Supplementary Table 3). The weak enhancement of CD8^+^ CTLs and presence of CD4^+^ cells suggest that these tumors were probably in early stages of active immune response compared to the mIL12 mRNA-treated Yummer B2M KO tumors. In contrast with Yummer B2M KO, CD4^+^CD25^+^ cells accounted for a relatively larger proportion, approximately 30% of the CD4 cells in the MC38 B2M KO tumors (Supplementary Table 3). Again, in contrast with the observation in treated Yummer B2M tumors, treatment with mIL12 mRNA or anti-PD-L1 did not significantly affect the numbers of CD4^+^CD69^+^CD25^−^ subtype cells but the population of the CD4^+^CD25^+^ cells was reduced by half suggesting that this population plays a regulatory role in this model. Combination treatment with anti-PD-L1 resulted in additional decreases compared to monotherapy (Fig. 5E and F, Supplementary Table 3).

Histological examination of Yummer B2M KO and MC38 B2M KO control tumors showed remarkable similarities. H&E stained sections from both these models demonstrated characteristics of rapidly dividing tumor cells, evidenced by prevalence of large nodules of epithelioid tumor cells with a significantly higher rate of mitotic events (Fig. 5G and H, Supplementary Fig. S4A). The mIL12 mRNA-treated Yummer 1.7 B2M KO tumors showed areas of extensive tumor cell death with sections from 3/5 tumors showing scarce tumor cell containing areas precluding extensive quantification of death events. The mIL12 mRNA-treated MC38 B2M KO tumors showed trends of increased occurrence of pyknotic events compared to controls (Supplementary Fig. 4B).

IHC analyses by CD3 staining of control treated tumor sections from both models showed presence of significant numbers of T cells. These T cells were mostly restricted to the tumor-infiltrating edge along the periphery of the tumor which is suggestive of a failed immune response (Fig. 5G and H). CD3 staining in both Yummer B2M KO and MC38 B2M KO mIL12 mRNA-treated tumor sections showed increased inflammatory responses in the periphery and a robust infiltration of T cells deep into the tumor and associated destruction of tumor cells (Fig. 5G and H).

## Discussion

There is an urgent, unmet need for new therapies to improve outcomes for cancer patients with ICI-resistant tumors. The previous work using the mouse version of the drug, mIL12 mRNA, demonstrated robust TGI and combination activity with anti-PD-L1 therapy [22, 27]. A T_H_1 type response and resultant remodeling of the innate and adaptive immune cells of TME to an activated state was determined to underlie the powerful tumor inhibition elicited by this approach [22].

Blunted antigen presentation due to MHCI deficiencies resulting from B2M loss of function is thought to be a major cause of ICI resistance in many human cancer types. B2M knockout syngeneic models have been shown previously to be resistant to ICI therapy and to possess an immune profile similar to ICI-resistant human cancers [23–25]. The two syngeneic B2M KO models utilized in this study demonstrated different tumor growth kinetics and baseline immune profiles. They also exhibited varying levels of resistance to ICI therapy. The Yummer B2M KO tumors were completely unresponsive to anti-PD-L1 treatment while the MC38 B2M KO tumors were not fully resistant and CRs occurred in a minority (20%) of treated mice. Interestingly, monotherapy with a single intratumoral injection of mIL12 mRNA was universally effective in both models; with powerful Th1 orientated immune responses detected in all treated animals. The high CR rates and failed establishment of tumors in rechallenged mice are indicative of durable systemic immunological memory achieved by mIL12 mRNA monotherapy even in a MHCI-deficient state.

Local administration of immune activating cytokines has been shown previously to be effective in inducing anti-tumor immune responses in ICI-resistant tumor models and the immune mechanisms that cause tumor regression varied based on the background models and the agents that were used [22, 28, 29]. Based on our observations in this study, we propose that localized expression of the recombinant mIL12 within the tumor microenvironment caused a shift of the TME from an immune suppressed to T_H_1 type activated state associated with a rise in levels of key immune activating and chemoattractant cytokines. Localized expression of the recombinant mIL12 resulted in significantly elevated numbers of activated TAMs in the TME of Yummer B2M KO tumors. Localized IL12 production and creation of an immune-permissive environment also appeared to reverse T-cell exclusion; with higher densities of CTLs detected throughout in the Yummer B2M KO tumors. These changes were less apparent in the MC38 B2M KO model, which had higher numbers of TAMs at baseline.

Combination therapies with pooled cytokines including IL12 have been previously described in an ICI-resistant mouse model obtained by serial passaging of PD-1 therapy escaping tumor cells. The tumors expressed MHCI did not possess genetic alterations commonly known to cause ICI resistance and the TME immunologically cold with low infiltration of T cells, NK cells and macrophages [28]. In another such study, syngeneic tumor models were treated with a pool of IL7, IL12 and IFNα [30]. The responses observed were relatively muted possibly due to alterations in multiple immune pathways in these models. Cytokine profiling data from, B2M KO tumors in our study show that mIL12 mRNA treatment is sufficient to cause significant upregulation of cytokines like IL7, GM-CSF and IL15 which were key constituents of the pooled cytokine strategies.

Elevation of IFNγ levels, which is indicative of favorable outcomes to immune therapies [31–33], occurred in all tumors that were administered mIL12 mRNA. Upregulation of multiple chemokines that promote attraction and infiltration of effector immune cells deep into the tumors was a key feature in both models. The chemokines CCL2 and CCL5 well known attractants of CTLs as well as CXCL9 and CXCL10 powerful chemoattractant of activated CTLs were all highly induced by mIL12 mRNA treatment in both models and likely laid the ground for trafficking and retention of these effectors deep within the tumor cores.

The balance of pro-tumor and anti-tumor TAMs is altered by mIL12 mRNA treatment and appears to be associated with baseline TAM infiltration and levels of the macrophage inducing cytokine CCL2. In the Yummer B2M KO melanomas, with relatively lesser TAM infiltration, compared to MC38 B2M KO tumors, a robust induction of CCL2 is observed along with a significant increase in TAM numbers. Both immune activating as well as suppressive macrophages are expanded in number; however, the suppressive types are a fraction of the activated macrophages. The macrophages are associated with histologically evident phagocytic clearance of tumor cells which is quite expected due to the concerted action of upregulated immune activating effects of IFNγ and TNFα. On the other hand, in the colorectal, MC38 B2M KO tumors, which at a baseline were already highly infiltrated by TAMs, and upon mIL12 mRNA treatment had a relatively lower induction of CCL2 levels compared to Yummer B2M KO tumors, there was no consistent increase in total numbers of TAMs or the activated types. Anti-PD-L1 treatment resulted in increases of TAMs and activated subtypes in 3/5 tumors which could explain responses with ICI monotherapy in some individuals in this model. A reduction in the numbers of immune suppressive type TAMs was observed across all treatment conditions, and additional work needs to be done to determine if the increase in activated TAM numbers accompanied with a reduction in suppressive ones is associated with the slightly improved outcome in the combination therapy of the MC38 B2M KO tumors.

Some studies have suggested an active role of NK cells in anti-tumor responses driven by IL12 therapies in MHCI-deficient models [23, 24]. In this study, we observed modest increases in NK cell numbers in a few individuals in mIL12 mRNA and anti-PD-L1 monotherapy groups in both B2M KO models. Interestingly, several individuals in both these models showed declines in NK cell numbers in the combination therapy arms. MHC class I-deficient tumors can escape NK cell immune surveillance by inducing a state of ‘anergy’ in these cells. IL12 cytokine treatment can restore signaling and reverse NK cell functional anergy resulting in effective tumor cell killing [34]. Further studies are merited to investigate the functional state of NK cells post-mIL12 mRNA treatment in these tumor models. These studies can also determine if decreased numbers upon combination treatment is responsible for longer times to CRs in both models and for the slightly worse outcome observed in the Yummer B2M KO model compared to mIL12 mRNA monotherapy.

The CD4 cells infiltrating the tumors in both models fit into two categories, CD4^+^CD69^+^CD25^−^ and CD4^+^CD25 + cells based on surface expression. The Yummer B2M KO tumors, infiltrating T helpers, were mostly of the CD69^+^CD25^−^ subtype, making up > 80% of the CD4s. These types of cells have been described to have a regulatory role in potent inhibition of T-cell proliferation in murine models and are known to be associated with relapse in human cancers [35, 36]. The numbers of these cells were decreased by over threefold upon treatment with mIL12 mRNA which would have allowed increased proliferation of cytotoxic T cells. In the MC38 B2M KO tumors, CD4^+^CD25^+^ cells were present in significant numbers along with the CD4^+^CD69^+^CD25^−^subtype. Unlike the Yummer B2M KO tumors, the CD4^+^CD25^+^ population decreased upon treatment both with mIL12 mRNA as well as anti-PD-L1 indicating that these cells played a suppressive, regulatory role in this model.

Generally, pharmacologically inhibiting CD4^+^ T cells has been shown to be associated with improved CD8^+^ cytotoxic responses in several syngeneic models, and exhaustion of CD4 T cells in the TME could dampen CTL responses [37–39].

Our observations have clearly shown that local administration of mIL12 mRNA into ICI-resistant murine tumors, kick starts a cascade of immune events within the TME including upregulation of activating cytokines and chemokines; activation and increase in numbers of innate immune cells such as TAMs and NK cells and reordering of the Treg, effector CTL balance. The concerted immune attack on tumor cells across multiple fronts results in powerful immune-mediated tumor inhibition and high CR rates.

In multiple, limited Phase I clinical trials, several groups have shown that IT injected recombinant human IL12 mRNAs demonstrate an acceptable safety profile as a monotherapy when given to patients with late-stage disease. In an ongoing clinical trial, patients with solid tumors who failed available treatments received IL12 mRNA as 4–5th line therapy. Mild treatment related adverse events, grade 1 or 2 occurred with no dose-limiting toxicities or treatment discontinuation. Ongoing stable disease and progression free survival were observed in a subset of the patients receiving therapy [40]. Different mRNA formulations are tolerated well at the highest doses administered and result in immune stimulatory effects in the TME including elevated expression of PD-L1 [40–43].

Multiple administrations of IL12 mRNA drug sequentially or concurrently with combination immunotherapy with anti-PD-L1 CPI, durvalumab were well tolerated for periods stretching to 2 years resulting in confirmed partial responses including at distant uninjected sites suggestive of abscopal, anti-tumor immune activation [43]. Our observations in these two independent MHCI-deficient models suggest that tumor localized IL12 mRNA treatment is a promising therapeutic approach for human patients with ICI resistant, advanced stage human cancers. Additionally, IL12 therapy has been shown to synergize with classic chemotherapy, radiation treatments in eradication of large established murine tumors [26, 44–46]. Further, adoptive cell therapies which do not depend on MHCI expression and innate immune pathway activating agents such as CD40, STING and TLR agonists have been shown in preclinical settings to rely on and benefit from activated IFN signaling [13, 23, 27]. As such, these observations imply that activity of these therapeutics could be enhanced by concurrent IL12 stimulation of IFNγ production immune effectors in the local tumor environment. This makes a strong case for consideration of combination therapy strategies to include administration of intratumoral IL12 mRNA in clinical trials across broad anti-cancer therapy approaches.

## Supplementary Information

Below is the link to the electronic supplementary material.

Supplementary file 1 (PPTX 641 KB)

Supplementary file 2 (PPTX 75 KB)

## Data Availability

No datasets were generated or analyzed during the current study.
