# Supplementary material for: Intratumoral IL12 mRNA administration activates innate and adaptive pathways in checkpoint inhibitor-resistant tumors resulting in complete responses
Source: Cancer Immunol Immunother. 2025 Jun 25;74(8):250. doi: 10.1007/s00262-025-04105-0 (PMC12198101; doi:10.1007/s00262-025-04105-0)
Supplement: Supplementary file 1 — (PPTX 641 KB) [file 262_2025_4105_MOESM1_ESM.pptx]

## Slide 1
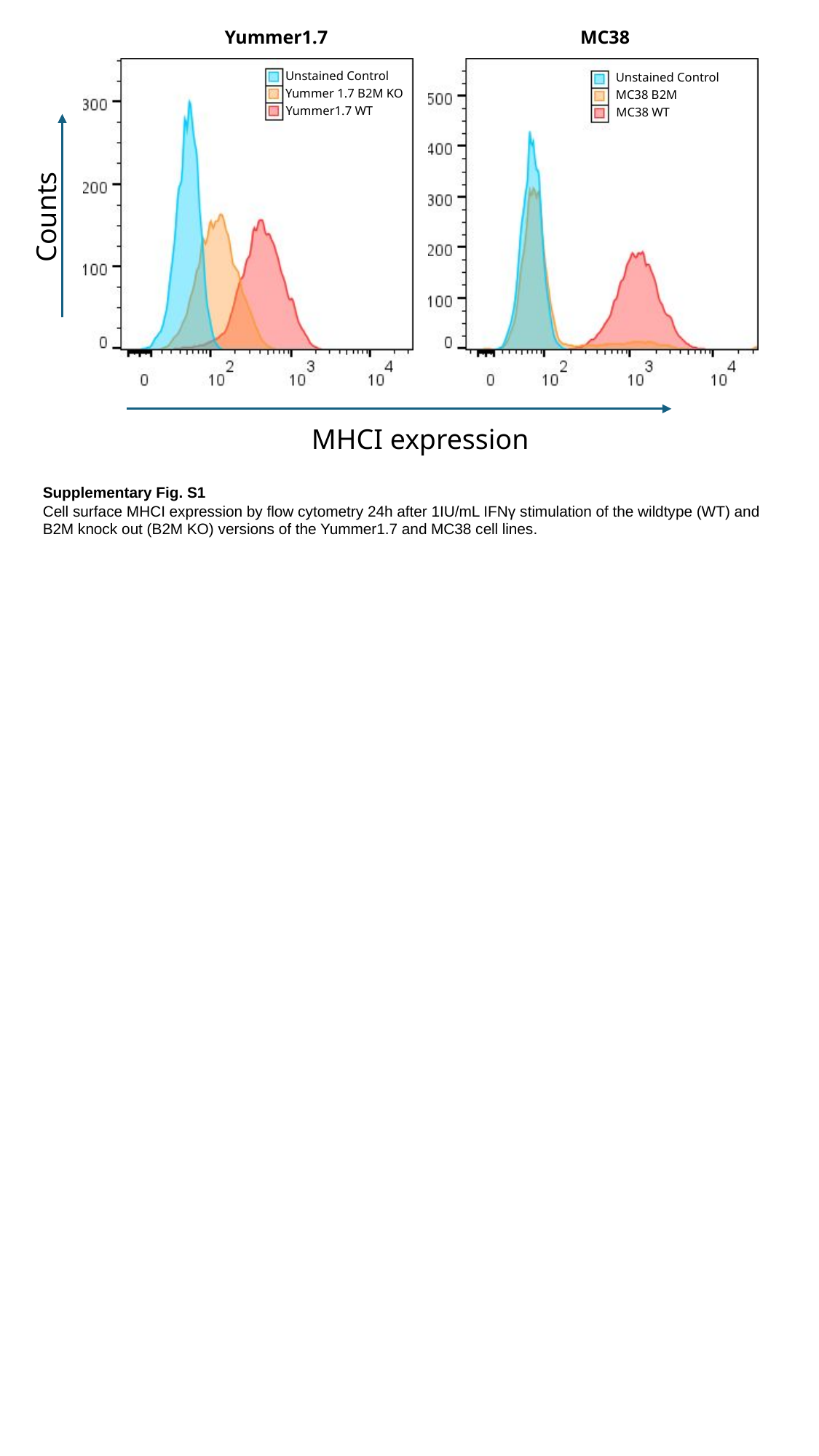

MC38
Yummer1.7
Counts
MHCI expression
Unstained Control
Unstained Control
Yummer 1.7 B2M KO
MC38 B2M
Yummer1.7 WT
MC38 WT
Supplementary Fig. S1
Cell surface MHCI expression by flow cytometry 24h after 1IU/mL IFNγ stimulation of the wildtype (WT) and B2M knock out (B2M KO) versions of the Yummer1.7 and MC38 cell lines.

## Slide 2
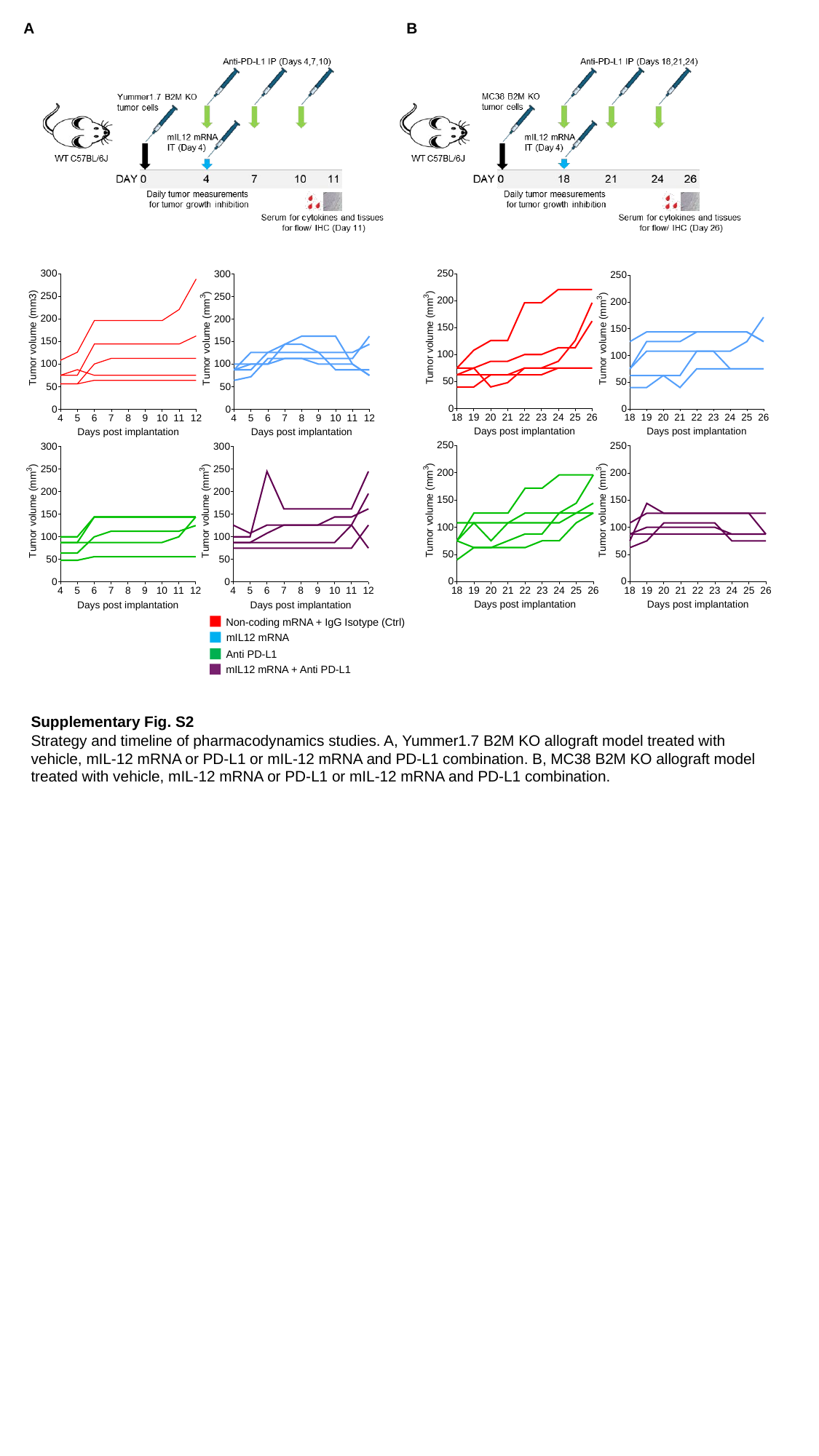

A
B
Non-coding mRNA + IgG Isotype (Ctrl)
mIL12 mRNA
Anti PD-L1
mIL12 mRNA + Anti PD-L1
Supplementary Fig. S2
Strategy and timeline of pharmacodynamics studies. A, Yummer1.7 B2M KO allograft model treated with vehicle, mIL-12 mRNA or PD-L1 or mIL-12 mRNA and PD-L1 combination. B, MC38 B2M KO allograft model treated with vehicle, mIL-12 mRNA or PD-L1 or mIL-12 mRNA and PD-L1 combination.

## Slide 3
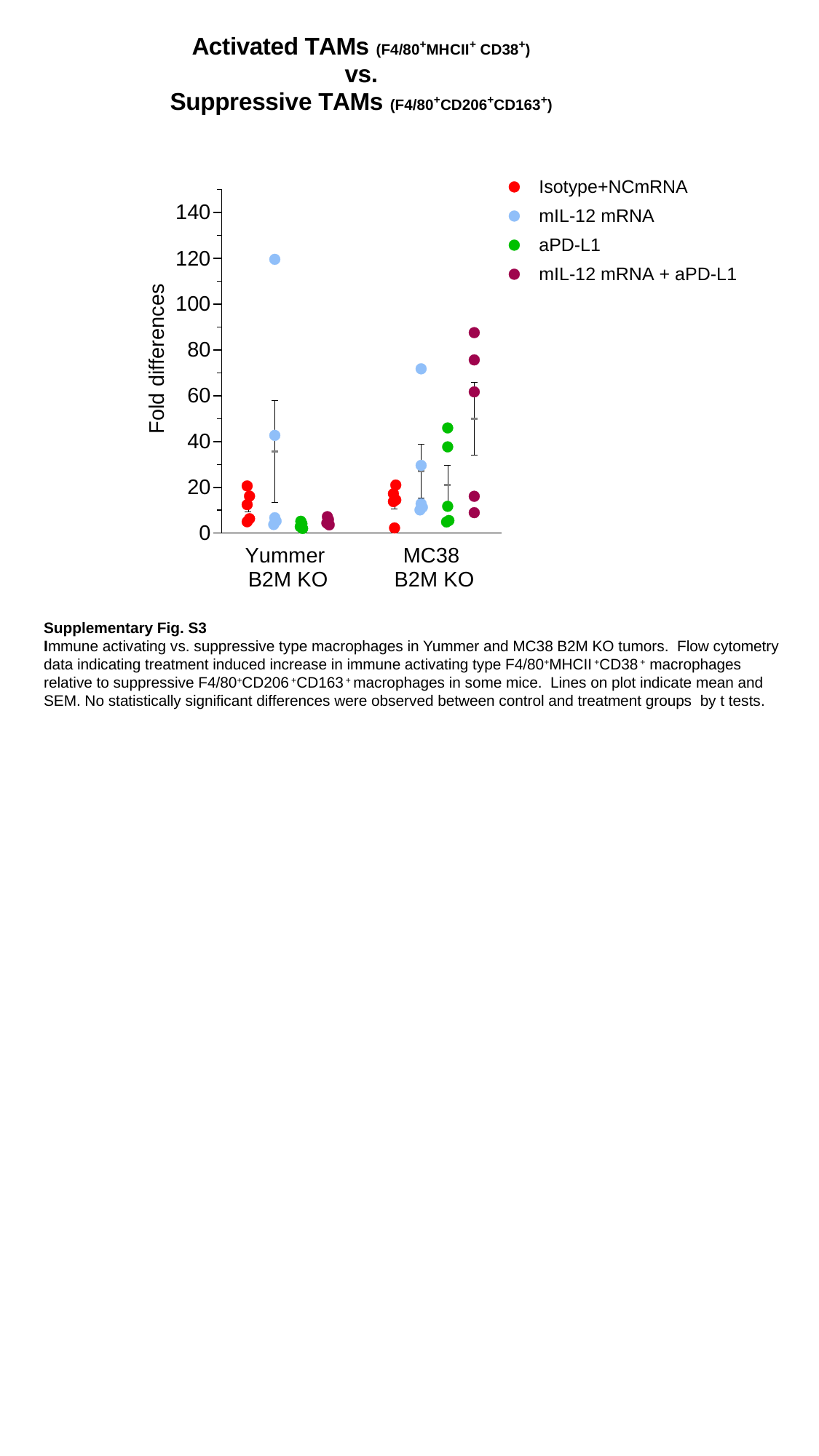

Supplementary Fig. S3
Immune activating vs. suppressive type macrophages in Yummer and MC38 B2M KO tumors. Flow cytometry data indicating treatment induced increase in immune activating type F4/80+MHCII +CD38 + macrophages relative to suppressive F4/80+CD206 +CD163 + macrophages in some mice. Lines on plot indicate mean and SEM. No statistically significant differences were observed between control and treatment groups by t tests.

## Slide 4
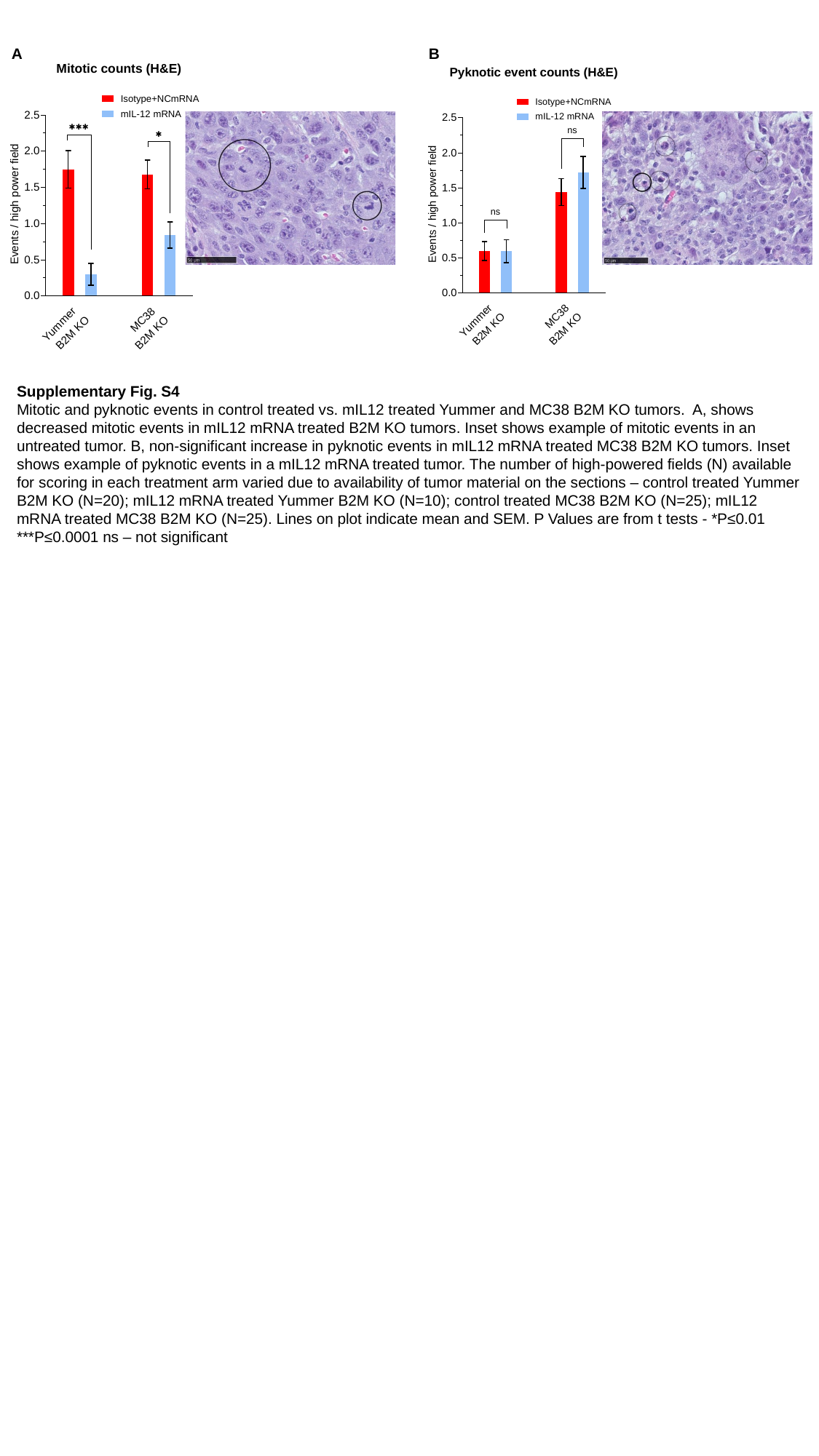

B
A
Supplementary Fig. S4
Mitotic and pyknotic events in control treated vs. mIL12 treated Yummer and MC38 B2M KO tumors. A, shows decreased mitotic events in mIL12 mRNA treated B2M KO tumors. Inset shows example of mitotic events in an untreated tumor. B, non-significant increase in pyknotic events in mIL12 mRNA treated MC38 B2M KO tumors. Inset shows example of pyknotic events in a mIL12 mRNA treated tumor. The number of high-powered fields (N) available for scoring in each treatment arm varied due to availability of tumor material on the sections – control treated Yummer B2M KO (N=20); mIL12 mRNA treated Yummer B2M KO (N=10); control treated MC38 B2M KO (N=25); mIL12 mRNA treated MC38 B2M KO (N=25). Lines on plot indicate mean and SEM. P Values are from t tests - *P≤0.01 ***P≤0.0001 ns – not significant
