# Supplementary material for: Intratumoral IL12 mRNA administration activates innate and adaptive pathways in checkpoint inhibitor-resistant tumors resulting in complete responses
Source: Cancer Immunol Immunother. 2025 Jun 25;74(8):250. doi: 10.1007/s00262-025-04105-0 (PMC12198101; doi:10.1007/s00262-025-04105-0)
Supplement: Supplementary file 2 — (PPTX 75 KB) [file 262_2025_4105_MOESM2_ESM.pptx]

## Slide 1
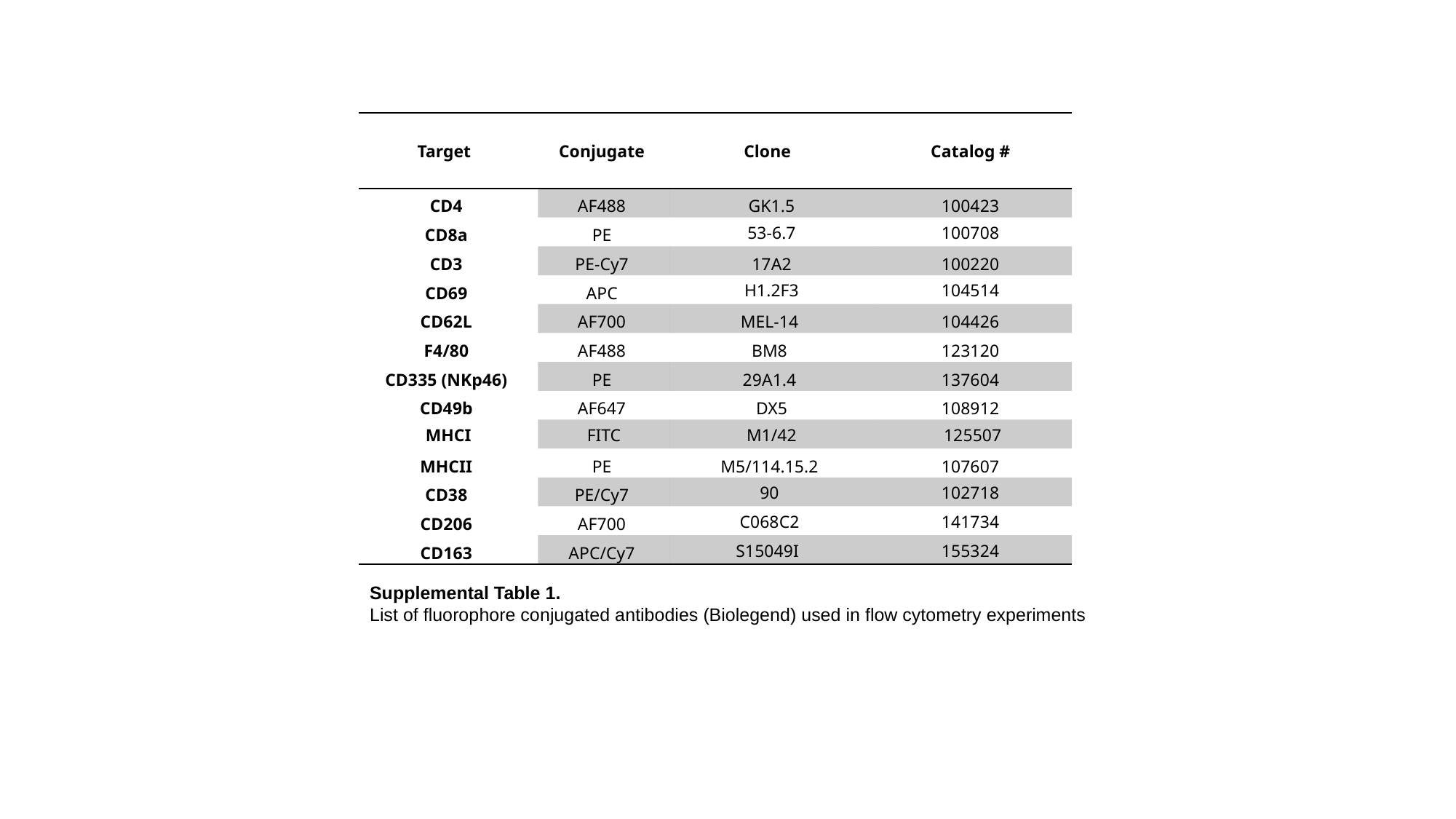

| Target | Conjugate | Clone | Catalog # |
| --- | --- | --- | --- |
| CD4 | AF488 | GK1.5 | 100423 |
| CD8a | PE | 53-6.7 | 100708 |
| CD3 | PE-Cy7 | 17A2 | 100220 |
| CD69 | APC | H1.2F3 | 104514 |
| CD62L | AF700 | MEL-14 | 104426 |
| F4/80 | AF488 | BM8 | 123120 |
| CD335 (NKp46) | PE | 29A1.4 | 137604 |
| CD49b | AF647 | DX5 | 108912 |
| MHCI | FITC | M1/42 | 125507 |
| MHCII | PE | M5/114.15.2 | 107607 |
| CD38 | PE/Cy7 | 90 | 102718 |
| CD206 | AF700 | C068C2 | 141734 |
| CD163 | APC/Cy7 | S15049I | 155324 |
Supplemental Table 1.
List of fluorophore conjugated antibodies (Biolegend) used in flow cytometry experiments

## Slide 2
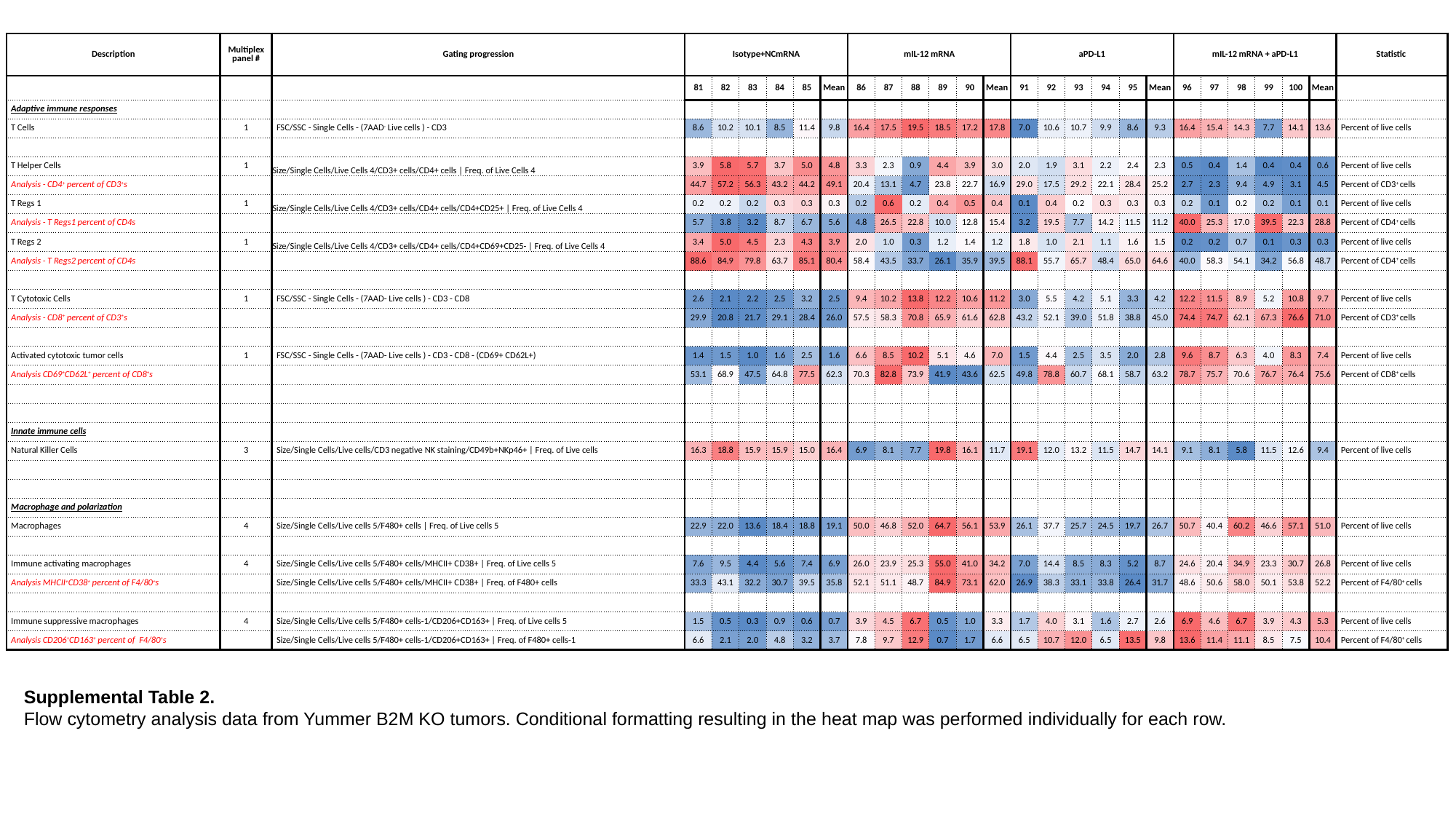

| Description | Multiplex panel # | Gating progression | Isotype+NCmRNA | | | | | | mIL-12 mRNA | | | | | | aPD-L1 | | | | | | mIL-12 mRNA + aPD-L1 | | | | | | Statistic |
| --- | --- | --- | --- | --- | --- | --- | --- | --- | --- | --- | --- | --- | --- | --- | --- | --- | --- | --- | --- | --- | --- | --- | --- | --- | --- | --- | --- |
| | | | 81 | 82 | 83 | 84 | 85 | Mean | 86 | 87 | 88 | 89 | 90 | Mean | 91 | 92 | 93 | 94 | 95 | Mean | 96 | 97 | 98 | 99 | 100 | Mean | |
| Adaptive immune responses | | | | | | | | | | | | | | | | | | | | | | | | | | | |
| T Cells | 1 | FSC/SSC - Single Cells - (7AAD- Live cells ) - CD3 | 8.6 | 10.2 | 10.1 | 8.5 | 11.4 | 9.8 | 16.4 | 17.5 | 19.5 | 18.5 | 17.2 | 17.8 | 7.0 | 10.6 | 10.7 | 9.9 | 8.6 | 9.3 | 16.4 | 15.4 | 14.3 | 7.7 | 14.1 | 13.6 | Percent of live cells |
| | | | | | | | | | | | | | | | | | | | | | | | | | | | |
| T Helper Cells | 1 | Size/Single Cells/Live Cells 4/CD3+ cells/CD4+ cells | Freq. of Live Cells 4 | 3.9 | 5.8 | 5.7 | 3.7 | 5.0 | 4.8 | 3.3 | 2.3 | 0.9 | 4.4 | 3.9 | 3.0 | 2.0 | 1.9 | 3.1 | 2.2 | 2.4 | 2.3 | 0.5 | 0.4 | 1.4 | 0.4 | 0.4 | 0.6 | Percent of live cells |
| Analysis - CD4+ percent of CD3+s | | | 44.7 | 57.2 | 56.3 | 43.2 | 44.2 | 49.1 | 20.4 | 13.1 | 4.7 | 23.8 | 22.7 | 16.9 | 29.0 | 17.5 | 29.2 | 22.1 | 28.4 | 25.2 | 2.7 | 2.3 | 9.4 | 4.9 | 3.1 | 4.5 | Percent of CD3+ cells |
| T Regs 1 | 1 | Size/Single Cells/Live Cells 4/CD3+ cells/CD4+ cells/CD4+CD25+ | Freq. of Live Cells 4 | 0.2 | 0.2 | 0.2 | 0.3 | 0.3 | 0.3 | 0.2 | 0.6 | 0.2 | 0.4 | 0.5 | 0.4 | 0.1 | 0.4 | 0.2 | 0.3 | 0.3 | 0.3 | 0.2 | 0.1 | 0.2 | 0.2 | 0.1 | 0.1 | Percent of live cells |
| Analysis - T Regs1 percent of CD4s | | | 5.7 | 3.8 | 3.2 | 8.7 | 6.7 | 5.6 | 4.8 | 26.5 | 22.8 | 10.0 | 12.8 | 15.4 | 3.2 | 19.5 | 7.7 | 14.2 | 11.5 | 11.2 | 40.0 | 25.3 | 17.0 | 39.5 | 22.3 | 28.8 | Percent of CD4+ cells |
| T Regs 2 | 1 | Size/Single Cells/Live Cells 4/CD3+ cells/CD4+ cells/CD4+CD69+CD25- | Freq. of Live Cells 4 | 3.4 | 5.0 | 4.5 | 2.3 | 4.3 | 3.9 | 2.0 | 1.0 | 0.3 | 1.2 | 1.4 | 1.2 | 1.8 | 1.0 | 2.1 | 1.1 | 1.6 | 1.5 | 0.2 | 0.2 | 0.7 | 0.1 | 0.3 | 0.3 | Percent of live cells |
| Analysis - T Regs2 percent of CD4s | | | 88.6 | 84.9 | 79.8 | 63.7 | 85.1 | 80.4 | 58.4 | 43.5 | 33.7 | 26.1 | 35.9 | 39.5 | 88.1 | 55.7 | 65.7 | 48.4 | 65.0 | 64.6 | 40.0 | 58.3 | 54.1 | 34.2 | 56.8 | 48.7 | Percent of CD4+ cells |
| | | | | | | | | | | | | | | | | | | | | | | | | | | | |
| T Cytotoxic Cells | 1 | FSC/SSC - Single Cells - (7AAD- Live cells ) - CD3 - CD8 | 2.6 | 2.1 | 2.2 | 2.5 | 3.2 | 2.5 | 9.4 | 10.2 | 13.8 | 12.2 | 10.6 | 11.2 | 3.0 | 5.5 | 4.2 | 5.1 | 3.3 | 4.2 | 12.2 | 11.5 | 8.9 | 5.2 | 10.8 | 9.7 | Percent of live cells |
| Analysis - CD8+ percent of CD3+s | | | 29.9 | 20.8 | 21.7 | 29.1 | 28.4 | 26.0 | 57.5 | 58.3 | 70.8 | 65.9 | 61.6 | 62.8 | 43.2 | 52.1 | 39.0 | 51.8 | 38.8 | 45.0 | 74.4 | 74.7 | 62.1 | 67.3 | 76.6 | 71.0 | Percent of CD3+ cells |
| | | | | | | | | | | | | | | | | | | | | | | | | | | | |
| Activated cytotoxic tumor cells | 1 | FSC/SSC - Single Cells - (7AAD- Live cells ) - CD3 - CD8 - (CD69+ CD62L+) | 1.4 | 1.5 | 1.0 | 1.6 | 2.5 | 1.6 | 6.6 | 8.5 | 10.2 | 5.1 | 4.6 | 7.0 | 1.5 | 4.4 | 2.5 | 3.5 | 2.0 | 2.8 | 9.6 | 8.7 | 6.3 | 4.0 | 8.3 | 7.4 | Percent of live cells |
| Analysis CD69+CD62L+ percent of CD8+s | | | 53.1 | 68.9 | 47.5 | 64.8 | 77.5 | 62.3 | 70.3 | 82.8 | 73.9 | 41.9 | 43.6 | 62.5 | 49.8 | 78.8 | 60.7 | 68.1 | 58.7 | 63.2 | 78.7 | 75.7 | 70.6 | 76.7 | 76.4 | 75.6 | Percent of CD8+ cells |
| | | | | | | | | | | | | | | | | | | | | | | | | | | | |
| | | | | | | | | | | | | | | | | | | | | | | | | | | | |
| Innate immune cells | | | | | | | | | | | | | | | | | | | | | | | | | | | |
| Natural Killer Cells | 3 | Size/Single Cells/Live cells/CD3 negative NK staining/CD49b+NKp46+ | Freq. of Live cells | 16.3 | 18.8 | 15.9 | 15.9 | 15.0 | 16.4 | 6.9 | 8.1 | 7.7 | 19.8 | 16.1 | 11.7 | 19.1 | 12.0 | 13.2 | 11.5 | 14.7 | 14.1 | 9.1 | 8.1 | 5.8 | 11.5 | 12.6 | 9.4 | Percent of live cells |
| | | | | | | | | | | | | | | | | | | | | | | | | | | | |
| | | | | | | | | | | | | | | | | | | | | | | | | | | | |
| Macrophage and polarization | | | | | | | | | | | | | | | | | | | | | | | | | | | |
| Macrophages | 4 | Size/Single Cells/Live cells 5/F480+ cells | Freq. of Live cells 5 | 22.9 | 22.0 | 13.6 | 18.4 | 18.8 | 19.1 | 50.0 | 46.8 | 52.0 | 64.7 | 56.1 | 53.9 | 26.1 | 37.7 | 25.7 | 24.5 | 19.7 | 26.7 | 50.7 | 40.4 | 60.2 | 46.6 | 57.1 | 51.0 | Percent of live cells |
| | | | | | | | | | | | | | | | | | | | | | | | | | | | |
| Immune activating macrophages | 4 | Size/Single Cells/Live cells 5/F480+ cells/MHCII+ CD38+ | Freq. of Live cells 5 | 7.6 | 9.5 | 4.4 | 5.6 | 7.4 | 6.9 | 26.0 | 23.9 | 25.3 | 55.0 | 41.0 | 34.2 | 7.0 | 14.4 | 8.5 | 8.3 | 5.2 | 8.7 | 24.6 | 20.4 | 34.9 | 23.3 | 30.7 | 26.8 | Percent of live cells |
| Analysis MHCII+CD38+ percent of F4/80+s | | Size/Single Cells/Live cells 5/F480+ cells/MHCII+ CD38+ | Freq. of F480+ cells | 33.3 | 43.1 | 32.2 | 30.7 | 39.5 | 35.8 | 52.1 | 51.1 | 48.7 | 84.9 | 73.1 | 62.0 | 26.9 | 38.3 | 33.1 | 33.8 | 26.4 | 31.7 | 48.6 | 50.6 | 58.0 | 50.1 | 53.8 | 52.2 | Percent of F4/80+ cells |
| | | | | | | | | | | | | | | | | | | | | | | | | | | | |
| Immune suppressive macrophages | 4 | Size/Single Cells/Live cells 5/F480+ cells-1/CD206+CD163+ | Freq. of Live cells 5 | 1.5 | 0.5 | 0.3 | 0.9 | 0.6 | 0.7 | 3.9 | 4.5 | 6.7 | 0.5 | 1.0 | 3.3 | 1.7 | 4.0 | 3.1 | 1.6 | 2.7 | 2.6 | 6.9 | 4.6 | 6.7 | 3.9 | 4.3 | 5.3 | Percent of live cells |
| Analysis CD206+CD163+ percent of F4/80+s | | Size/Single Cells/Live cells 5/F480+ cells-1/CD206+CD163+ | Freq. of F480+ cells-1 | 6.6 | 2.1 | 2.0 | 4.8 | 3.2 | 3.7 | 7.8 | 9.7 | 12.9 | 0.7 | 1.7 | 6.6 | 6.5 | 10.7 | 12.0 | 6.5 | 13.5 | 9.8 | 13.6 | 11.4 | 11.1 | 8.5 | 7.5 | 10.4 | Percent of F4/80+ cells |
Supplemental Table 2.
Flow cytometry analysis data from Yummer B2M KO tumors. Conditional formatting resulting in the heat map was performed individually for each row.

## Slide 3
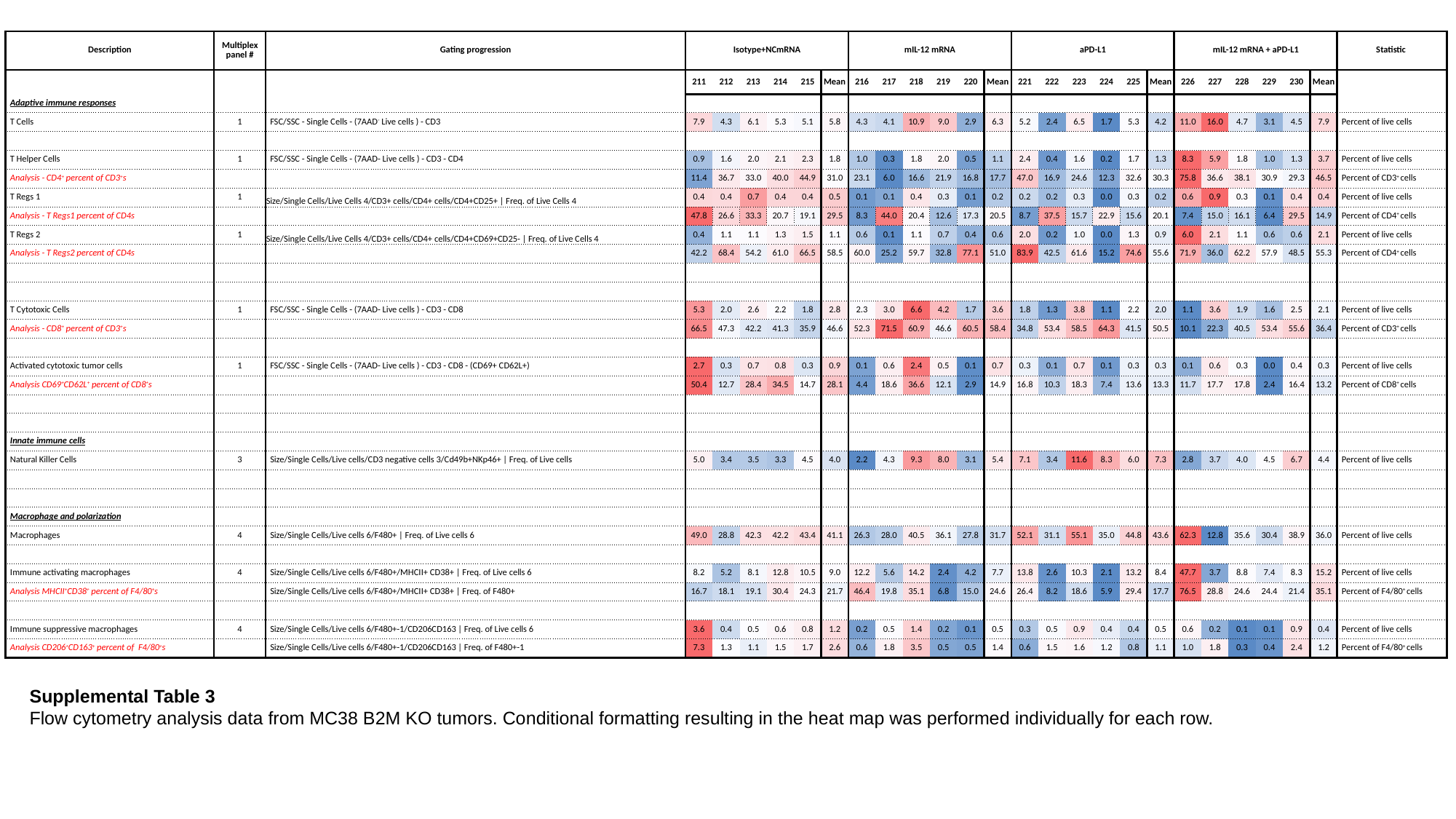

| Description | Multiplex panel # | Gating progression | Isotype+NCmRNA | | | | | | mIL-12 mRNA | | | | | | aPD-L1 | | | | | | mIL-12 mRNA + aPD-L1 | | | | | | Statistic |
| --- | --- | --- | --- | --- | --- | --- | --- | --- | --- | --- | --- | --- | --- | --- | --- | --- | --- | --- | --- | --- | --- | --- | --- | --- | --- | --- | --- |
| | | | 211 | 212 | 213 | 214 | 215 | Mean | 216 | 217 | 218 | 219 | 220 | Mean | 221 | 222 | 223 | 224 | 225 | Mean | 226 | 227 | 228 | 229 | 230 | Mean | |
| Adaptive immune responses | | | | | | | | | | | | | | | | | | | | | | | | | | | |
| T Cells | 1 | FSC/SSC - Single Cells - (7AAD- Live cells ) - CD3 | 7.9 | 4.3 | 6.1 | 5.3 | 5.1 | 5.8 | 4.3 | 4.1 | 10.9 | 9.0 | 2.9 | 6.3 | 5.2 | 2.4 | 6.5 | 1.7 | 5.3 | 4.2 | 11.0 | 16.0 | 4.7 | 3.1 | 4.5 | 7.9 | Percent of live cells |
| | | | | | | | | | | | | | | | | | | | | | | | | | | | |
| T Helper Cells | 1 | FSC/SSC - Single Cells - (7AAD- Live cells ) - CD3 - CD4 | 0.9 | 1.6 | 2.0 | 2.1 | 2.3 | 1.8 | 1.0 | 0.3 | 1.8 | 2.0 | 0.5 | 1.1 | 2.4 | 0.4 | 1.6 | 0.2 | 1.7 | 1.3 | 8.3 | 5.9 | 1.8 | 1.0 | 1.3 | 3.7 | Percent of live cells |
| Analysis - CD4+ percent of CD3+s | | | 11.4 | 36.7 | 33.0 | 40.0 | 44.9 | 31.0 | 23.1 | 6.0 | 16.6 | 21.9 | 16.8 | 17.7 | 47.0 | 16.9 | 24.6 | 12.3 | 32.6 | 30.3 | 75.8 | 36.6 | 38.1 | 30.9 | 29.3 | 46.5 | Percent of CD3+ cells |
| T Regs 1 | 1 | Size/Single Cells/Live Cells 4/CD3+ cells/CD4+ cells/CD4+CD25+ | Freq. of Live Cells 4 | 0.4 | 0.4 | 0.7 | 0.4 | 0.4 | 0.5 | 0.1 | 0.1 | 0.4 | 0.3 | 0.1 | 0.2 | 0.2 | 0.2 | 0.3 | 0.0 | 0.3 | 0.2 | 0.6 | 0.9 | 0.3 | 0.1 | 0.4 | 0.4 | Percent of live cells |
| Analysis - T Regs1 percent of CD4s | | | 47.8 | 26.6 | 33.3 | 20.7 | 19.1 | 29.5 | 8.3 | 44.0 | 20.4 | 12.6 | 17.3 | 20.5 | 8.7 | 37.5 | 15.7 | 22.9 | 15.6 | 20.1 | 7.4 | 15.0 | 16.1 | 6.4 | 29.5 | 14.9 | Percent of CD4+ cells |
| T Regs 2 | 1 | Size/Single Cells/Live Cells 4/CD3+ cells/CD4+ cells/CD4+CD69+CD25- | Freq. of Live Cells 4 | 0.4 | 1.1 | 1.1 | 1.3 | 1.5 | 1.1 | 0.6 | 0.1 | 1.1 | 0.7 | 0.4 | 0.6 | 2.0 | 0.2 | 1.0 | 0.0 | 1.3 | 0.9 | 6.0 | 2.1 | 1.1 | 0.6 | 0.6 | 2.1 | Percent of live cells |
| Analysis - T Regs2 percent of CD4s | | | 42.2 | 68.4 | 54.2 | 61.0 | 66.5 | 58.5 | 60.0 | 25.2 | 59.7 | 32.8 | 77.1 | 51.0 | 83.9 | 42.5 | 61.6 | 15.2 | 74.6 | 55.6 | 71.9 | 36.0 | 62.2 | 57.9 | 48.5 | 55.3 | Percent of CD4+ cells |
| | | | | | | | | | | | | | | | | | | | | | | | | | | | |
| | | | | | | | | | | | | | | | | | | | | | | | | | | | |
| T Cytotoxic Cells | 1 | FSC/SSC - Single Cells - (7AAD- Live cells ) - CD3 - CD8 | 5.3 | 2.0 | 2.6 | 2.2 | 1.8 | 2.8 | 2.3 | 3.0 | 6.6 | 4.2 | 1.7 | 3.6 | 1.8 | 1.3 | 3.8 | 1.1 | 2.2 | 2.0 | 1.1 | 3.6 | 1.9 | 1.6 | 2.5 | 2.1 | Percent of live cells |
| Analysis - CD8+ percent of CD3+s | | | 66.5 | 47.3 | 42.2 | 41.3 | 35.9 | 46.6 | 52.3 | 71.5 | 60.9 | 46.6 | 60.5 | 58.4 | 34.8 | 53.4 | 58.5 | 64.3 | 41.5 | 50.5 | 10.1 | 22.3 | 40.5 | 53.4 | 55.6 | 36.4 | Percent of CD3+ cells |
| | | | | | | | | | | | | | | | | | | | | | | | | | | | |
| Activated cytotoxic tumor cells | 1 | FSC/SSC - Single Cells - (7AAD- Live cells ) - CD3 - CD8 - (CD69+ CD62L+) | 2.7 | 0.3 | 0.7 | 0.8 | 0.3 | 0.9 | 0.1 | 0.6 | 2.4 | 0.5 | 0.1 | 0.7 | 0.3 | 0.1 | 0.7 | 0.1 | 0.3 | 0.3 | 0.1 | 0.6 | 0.3 | 0.0 | 0.4 | 0.3 | Percent of live cells |
| Analysis CD69+CD62L+ percent of CD8+s | | | 50.4 | 12.7 | 28.4 | 34.5 | 14.7 | 28.1 | 4.4 | 18.6 | 36.6 | 12.1 | 2.9 | 14.9 | 16.8 | 10.3 | 18.3 | 7.4 | 13.6 | 13.3 | 11.7 | 17.7 | 17.8 | 2.4 | 16.4 | 13.2 | Percent of CD8+ cells |
| | | | | | | | | | | | | | | | | | | | | | | | | | | | |
| | | | | | | | | | | | | | | | | | | | | | | | | | | | |
| Innate immune cells | | | | | | | | | | | | | | | | | | | | | | | | | | | |
| Natural Killer Cells | 3 | Size/Single Cells/Live cells/CD3 negative cells 3/Cd49b+NKp46+ | Freq. of Live cells | 5.0 | 3.4 | 3.5 | 3.3 | 4.5 | 4.0 | 2.2 | 4.3 | 9.3 | 8.0 | 3.1 | 5.4 | 7.1 | 3.4 | 11.6 | 8.3 | 6.0 | 7.3 | 2.8 | 3.7 | 4.0 | 4.5 | 6.7 | 4.4 | Percent of live cells |
| | | | | | | | | | | | | | | | | | | | | | | | | | | | |
| | | | | | | | | | | | | | | | | | | | | | | | | | | | |
| Macrophage and polarization | | | | | | | | | | | | | | | | | | | | | | | | | | | |
| Macrophages | 4 | Size/Single Cells/Live cells 6/F480+ | Freq. of Live cells 6 | 49.0 | 28.8 | 42.3 | 42.2 | 43.4 | 41.1 | 26.3 | 28.0 | 40.5 | 36.1 | 27.8 | 31.7 | 52.1 | 31.1 | 55.1 | 35.0 | 44.8 | 43.6 | 62.3 | 12.8 | 35.6 | 30.4 | 38.9 | 36.0 | Percent of live cells |
| | | | | | | | | | | | | | | | | | | | | | | | | | | | |
| Immune activating macrophages | 4 | Size/Single Cells/Live cells 6/F480+/MHCII+ CD38+ | Freq. of Live cells 6 | 8.2 | 5.2 | 8.1 | 12.8 | 10.5 | 9.0 | 12.2 | 5.6 | 14.2 | 2.4 | 4.2 | 7.7 | 13.8 | 2.6 | 10.3 | 2.1 | 13.2 | 8.4 | 47.7 | 3.7 | 8.8 | 7.4 | 8.3 | 15.2 | Percent of live cells |
| Analysis MHCII+CD38+ percent of F4/80+s | | Size/Single Cells/Live cells 6/F480+/MHCII+ CD38+ | Freq. of F480+ | 16.7 | 18.1 | 19.1 | 30.4 | 24.3 | 21.7 | 46.4 | 19.8 | 35.1 | 6.8 | 15.0 | 24.6 | 26.4 | 8.2 | 18.6 | 5.9 | 29.4 | 17.7 | 76.5 | 28.8 | 24.6 | 24.4 | 21.4 | 35.1 | Percent of F4/80+ cells |
| | | | | | | | | | | | | | | | | | | | | | | | | | | | |
| Immune suppressive macrophages | 4 | Size/Single Cells/Live cells 6/F480+-1/CD206CD163 | Freq. of Live cells 6 | 3.6 | 0.4 | 0.5 | 0.6 | 0.8 | 1.2 | 0.2 | 0.5 | 1.4 | 0.2 | 0.1 | 0.5 | 0.3 | 0.5 | 0.9 | 0.4 | 0.4 | 0.5 | 0.6 | 0.2 | 0.1 | 0.1 | 0.9 | 0.4 | Percent of live cells |
| Analysis CD206+CD163+ percent of F4/80+s | | Size/Single Cells/Live cells 6/F480+-1/CD206CD163 | Freq. of F480+-1 | 7.3 | 1.3 | 1.1 | 1.5 | 1.7 | 2.6 | 0.6 | 1.8 | 3.5 | 0.5 | 0.5 | 1.4 | 0.6 | 1.5 | 1.6 | 1.2 | 0.8 | 1.1 | 1.0 | 1.8 | 0.3 | 0.4 | 2.4 | 1.2 | Percent of F4/80+ cells |
Supplemental Table 3
Flow cytometry analysis data from MC38 B2M KO tumors. Conditional formatting resulting in the heat map was performed individually for each row.
